# RBX1 regulates *PKM* alternative splicing to facilitate anaplastic thyroid carcinoma metastasis and aerobic glycolysis by destroying the SMAR1/HDAC6 complex

**DOI:** 10.1186/s13578-023-00987-8

**Published:** 2023-02-21

**Authors:** Debin Xu, Jichun Yu, Yuting Yang, Yunyan Du, Hongcheng Lu, Shouhua Zhang, Qian Feng, Yi Yu, Liang Hao, Jun Shao, Leifeng Chen

**Affiliations:** 1 Department of Thyroid Surgery, Second Affiliated Hospital of Nanchang University, No. 1 Minde Road, Nanchang, 330008 China; 2 Department of Intensive Care Unit, First Affiliated Hospital of Nanchang University, No. 17, Yongwai Main Street, Nanchang, 330006 China; 3 School of Pharmacy, Nanchang University, No. 471, Bayi Road, Nanchang, 330006 China; 4 Department of General Surgery, Second Affiliated Hospital of Nanchang University, No. 1 Minde Road, Nanchang, 330008 China; 5 Department of General Surgery, Affiliated Children’s Hospital of Nanchang University, No. 122, Yangming Road, Nanchang, 330006 China; 6 Department of Urology, Second Affiliated Hospital of Nanchang University, No. 1 Minde Road, Nanchang, 330008 China; 7 Department of Orthopaedics, Second Affiliated Hospital of Nanchang University, No. 1 Minde Road, Nanchang, 330008 China; 8 Department of Cardiovascular Surgery, Second Affiliated Hospital of Nanchang University, No. 1 Minde Road, Nanchang, 330008 China; 9 Cancer Center, Renmin Hospital of Wuhan University, No. 238 Jiefang Road, Wuhan, 430060 China

**Keywords:** Anaplastic thyroid carcinoma, RBX1, Metastasis, PKM2, Aerobic glycolysis

## Abstract

**Background:**

Anaplastic thyroid carcinoma (ATC) is one of the most aggressive malignancies, frequently accompanied by metastasis and aerobic glycolysis. Cancer cells adjust their metabolism by modulating the *PKM* alternative splicing and facilitating PKM2 isoform expression. Therefore, identifying factors and mechanisms that control *PKM* alternative splicing is significant for overcoming the current challenges in ATC treatment.

**Results:**

In this study, the expression of RBX1 was largely enhanced in the ATC tissues. Our clinical tests suggested that high RBX1 expression was significantly related to poor survival. The functional analysis indicated that RBX1 facilitated the metastasis of ATC cells by enhancing the Warburg effect, and PKM2 played a key role in RBX1-mediated aerobic glycolysis. Furthermore, we confirmed that RBX1 regulates *PKM* alternative splicing and promotes the PKM2-mediated Warburg effect in ATC cells. Moreover, ATC cell migration and aerobic glycolysis induced by RBX1-mediated *PKM* alternative splicing are dependent on the destruction of the SMAR1/HDAC6 complex. RBX1, as an E3 ubiquitin ligase, degrades SMAR1 in ATC through the ubiquitin–proteasome pathway.

**Conclusion:**

Overall, our study identified the mechanism underlying the regulation of *PKM* alternative splicing in ATC cells for the first time and provides evidence about the effect of RBX1 on cellular adaptation to metabolic stress.

**Supplementary Information:**

The online version contains supplementary material available at 10.1186/s13578-023-00987-8.

## Background

Thyroid cancer is the most common cancer [1]. Papillary thyroid cancer (PTC) accounts for 85% of all thyroid cancer cases, and over 90% of people diagnosed with PTC can be cured [2]. On the contrary, anaplastic thyroid carcinoma (ATC) is the most malignant thyroid cancer, accounting for about 40% of thyroid cancer deaths [3]. ATC is one of the most rapidly progressing and aggressive cancers in contrast to PTC or other thyroid cancers [4]. Understanding the mechanisms of their metastasis and development is critical to developing therapeutic approaches for the treatment of ATC.

Oncogenic reprogramming of cellular metabolism is a critical feature of cancer cells [5, 6]. Aerobic glycolysis, also known as the Warburg effect, plays a significant role in the progression of several cancers, including non-small cell lung, liver, breast, thyroid, and bladder cancers [7]. Pyruvate kinase is a type of rate-limiting glycolytic enzyme that facilitates the phosphoenolpyruvate conversion to pyruvate [8]. It possesses four isoforms, namely, PKR, PKL, PKM2, and PKM1 [9]. Cancer cells can flexibly modulate gene expression at the level of alternative splicing to withstand hostile conditions. PKM1 and PKM2, two alternative splicing variants of the *PKM* gene, exhibited a switch in drug-resistant cancer cells, through the usage of mutually exclusive exons [10, 11]. PKM2 is typically expressed in cancer cells where it confers oncogenic features [12, 13]. Recent reports have highlighted the involvement of PKM2 in carcinogenesis, aerobic glycolysis, and metastasis of multiple cancers [14]. It is well-known that PKMs, especially PKM2, are overexpressed in some cancers. For example, PKM2 is overexpressed in more than 40% of patients with acute leukemia and is associated with adverse treatment outcomes [15]. In gastric cancer, PKM2 overexpression is associated with prognosis, indicating its potential role as a prognostic biomarker [16]. Cancer cells reprogramme their metabolism by inhibiting PKM2 and enhancing PDC activity, which increased anticancer effects in several types of cancer. Simultaneously, several studies have revealed that cancer cells arise from normal cells by rewiring their metabolism via modulating the alternative pre-mRNA splicing of *PKM* to promote PKM2 expression. Therefore, targeting this alternative splicing step is shown to be an efficacious strategy for the elimination of cancer cells [17, 18]. Recently, it has been shown that tumor suppressor SMAR1 regulates *PKM* alternative splicing by HDAC6-mediated deacetylation of PTBP1 [19]. Therefore, identification of the factors and mechanisms underlying *PKM* alternative splicing, resulting in the PKM2-mediated Warburg effect, is crucial for overcoming the current challenges in cancer treatment.

Protein modification by ubiquitin-like and ubiquitin proteins is a critical post-translational regulatory mechanism participating in cell physiology [20, 21]. The Skp1/Cullin/F-box (SCF) complex is the largest E3 ubiquitin ligase and a widely ubiquitinated protein [22, 23]. RBX1 was first isolated and purified from yeast as a significant component of the SCF complexes in yeast and human [22, 23]. Increasing evidence has shown that RBX1 expression promotes tumor progression and metastasis in several cancers, including non-small cell lung, liver, breast, ovarian, and bladder cancers [24–27]. Recently, it has been shown that RBX1 is a major regulator of the ubiquitin–proteasome system (UPS) in cancer [28, 29]. RBX1 facilitates the target protein ubiquitination carried out by ubiquitin ligase E3 and tags proteins for degradation [27, 30]. However, the exact role of RBX1 in ATC progression and the potential signaling cascade is still uncertain.

In this study, we confirmed that high levels of RBX1 expression are related to poor prognosis in ATC patients and revealed the molecular mechanisms underlying the impact of RBX1 on the metabolism and metastasis of ATC.

## Methods

### Patients and specimens

The tissue samples from 30 ATC patients were acquired from the Department of General Surgery of the Second Affiliated Hospital of Nanchang University. Pathologists verified that all the specimens were obtained from normal tissue. This study was approved by the Clinical Research Ethics Committee of the Second Affiliated Hospital of Nanchang University, and all subjects provided consent for research participation.

### Cell culture

Procell Life Science & Technology Co., Ltd. provided TPC-1, BCPAP, KTC-1, C643, CAL62, 8505C, KMH-2, KMH-5M, and BHT101. Cell lines were cultured in PRMI Medium 1640 or DMEM (Gibco; Thermo Fisher Scientific, Inc.) added with 10% fetal bovine serum (FBS) at 37 °C in a humidified CO_2_ incubator (5% CO_2_).

### Western blotting

RIPA buffer (Millipore; Sigma-Aldrich) was used for the lysis of ATC cells in accordance with the manufacturer’s instructions. BCA protein detection kit (Takara Biotechnology Co., Ltd) was used for measuring the total protein concentrations. 10% SDS-PAGE was used for the isolation of 20 µg samples, which were subsequently transferred to the PVDF membrane. These membranes were blocked by skim milk (5%) for 120 min at RT and subsequently incubated overnight at a temperature of 4 °C with one of the primary antibodies raised against RBX1 (1:10 00; cat. No. ab221548, Abcam), PKM (1:500; cat. No. ab150377, Abcam), ubiquitin (1:1000; cat. No. 10201-2-AP; Proteintech), SMAR1 (1:500; cat. No. Pa5-100402; Thermo Fisher Scientific), or HDAC6 (1:500; cat. No. ab133493; Abcam). Tubulin (1:2000; cat. No. 11224-1-ap; Proteintech Group, Inc.) was used as a loading control. The membranes were inoculated with the goat anti-rabbit IgG secondary antibody labeled with HRP (1:5000; cat. No. ab6728; Abcam) at a temperature of 4 °C after washing them thrice using Tris-buffered saline with 0.1% Tween 20. Finally, the ECL system and ImageJ software were used for visualizing and analyzing the protein bands, respectively.

### Immunohistochemistry (IHC)

Graded alcohol and xylene were used for treating the ATC tissue sections. Subsequently, the antigen retrieval was conducted in citrate buffer (0.01 M). The sections were blocked for half an hour in serum-free protein-blocking buffer, after which they were inoculated with anti-RBX1 (1:1,000; cat. no. ab221548, Abcam). The images were obtained and digitized using an Olympus BH-2 microscope (Olympus Corporation), and ImageJ 1.51v software (National Institutes of Health) was used for quantifying the DAB signals.

### shRNA

Short hairpin RNA (shRNA) used for the silencing of *RBX1* (shRBX1), *SMAR1* (shSMAR1), or *PKM2* (shPKM2) was produced by a company in Shanghai, China. The full-length human *PKM2, SMAR1*, and *RBX1* cDNAs were constructed using the genetic techniques and then ligated into pcDNA3.1 vector for producing p-PKM2, p-SMAR1, and HA-RBX1, respectively. A blank vector served as the negative control. Lipofectamine 3000 transfection reagent (Invitrogen, USA) was used to transfect these shRNA constructs and plasmids following the manufacturer’s instructions.

### Ubiquitination assay

CAL62 and ATC cells were transfected with Myc-Ub and/or Flag-SMAR1 expression plasmids with or without a combination of the plasmid encoding RBX1. The transfected cells were incubated for 30 h with or without exposure to MG132 (15 mmol/L) for 4 h before harvesting. The cell lysate was immunoprecipitated with a GST antibody, and the ubiquitination of SMAR1 was detected with an anti-FLAG antibody.

### Dual reporter PKM minigene assay

The cloning of dual reporter PKM minigene system and dual reporter PKM minigene assay were performed as described previously [19]. Mean ± SD fold change in eGFP/mCherry ratio was calculated for each sample and compared with the control sample.

### Migration and invasion assays

The invasiveness and migration of ATC cell lines were evaluated using Transwell assays with slight modifications. A layer of matrix gel is pre-coated on the upper portion of the polycarbonate membrane for the invasion test.

### In vivo metastasis assay

1 × 10^6^ cells in 100 μL phosphate-buffered saline were injected subcutaneously into the abdomen of nude mice. Once the diameter of the producing tumors reached 1–2 cm, the tumors were excised and then cut into pieces of about 1 mm^3^. These pieces were subsequently implanted into the nude mice (six in each group, male BALB/c^*nu/nu*^, from 6 to 8 weeks). These mice were euthanized 6 weeks after tumor implantation. Then, the lungs were excised, treated, and finally embedded in the paraffin. All animal studies were approved by the Animal Experimental Ethics Committee of the Second Affiliated Hospital of Nanchang University and were performed in accordance with the NIH Guide for the Care and Use of Experimental Animals.

### Measurement of extracellular acidification rate (ECAR) and oxygen consumption rate (OCR)

The cellular mitochondrial respiration and glycolytic capacity were detected by the extracellular flux analyzer XF96 (Seahorse Bioscience, Billerica, MA, USA). The Seahorse Bioscience analyzer and the XF cellular hydrodynamic test kit were applied separately following the manufacturer’s instructions.

### Co-immunoprecipitation (Co-IP) and ubiquitination assays

Immunoprecipitation was conducted as previously described [31]. For the ubiquitination assays in vivo, the ATC cells with the knockdown or overexpression of RBX1 were exposed to MG132 for four hours before harvest. After that, cell lysates were immunoprecipitated with an anti-SMAR1 antibody; while an anti-Ub antibody was used for identifying the ubiquitination of SMAR1.

### Statistical analysis

The data were presented as the mean ± SD of three separate tests. The differences among two groups and multiple groups were assessed using unpaired Student’s t-test and the ANOVA with posthoc Tukey test, respectively. The relationship between the clinicopathological features and the RBX1 expression of the ATC patients was analyzed using Fisher's exact test. SPSS 21.0 (IBM Corp.) in combination with GraphPad Prism 8.0 (GraphPad Software, Inc.) was employed for all the statistical calculations and the plotting of all the graphs. A statistical significance was considered when the *P* value was lower than 0.05. Experiments were conducted in triplicate with at least three biological replicates except for in vivo murine experiments which were two independent replicates.

## Results

### RBX1 overexpression is correlated with poor ATC prognosis

**Fig. 1:**
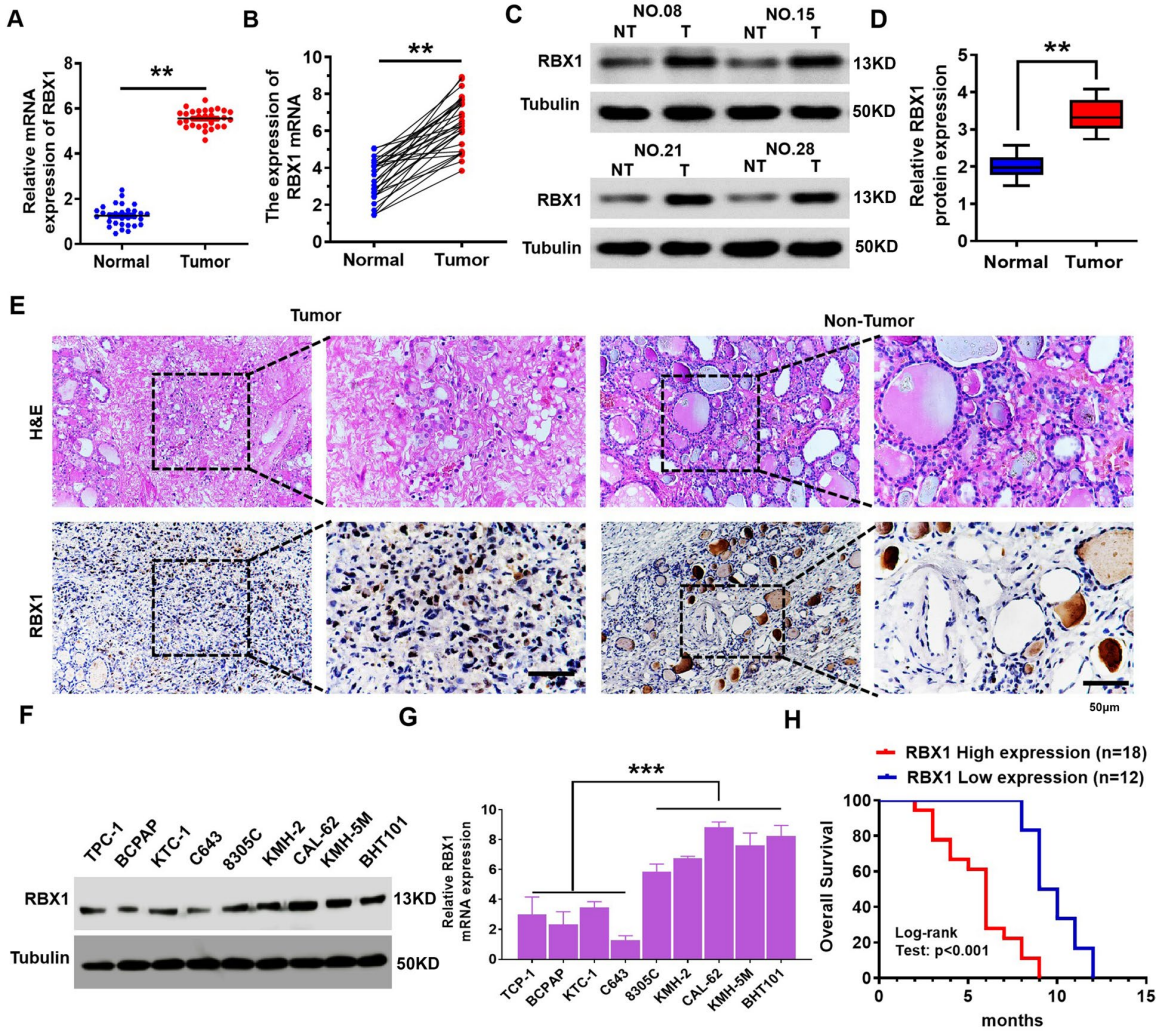
RBX1 is overexpressed in ATC and closely correlated with poor prognosis in patients. **A**, **B** *RBX1* mRNA expression levels in the ATC tissues together with the normal tissues adjacent to the tumor were investigated by qRT-PCR. **P < 0.01. **C**, **D** Expression levels of RBX1 protein in ATC tissues and the normal tissues adjacent to the tumor were investigated by western blotting. **P < 0.01. **E** RBX1 protein expression in the ATC tissues and the normal tissues adjacent to the tumor were determined using immunohistochemistry. Scale bar, 50 μm. **F**, **G** RBX1 protein and mRNA levels in ATC and PTC cell lines. ***P < 0.001. **H** Kaplan–Meier curves were used to visualize the overall survival of both low and high RBX1 expression of ATC patients. ***P < 0.001

To understand the clinical relevance of RBX1 to ATC, we measured the RBX1 expression in 30 ATC tissue samples together with corresponding adjacent tissues using western blotting and qRT-PCR. *RBX1* mRNA was overexpressed in tumor samples compared with their non-neoplastic counterparts (Fig. 1A, B). Furthermore, the levels of RBX1 protein were markedly elevated in ATC tissues, which was in agreement with the qRT-PCR results (Fig. 1C, D). As shown in Fig. 1E, RBX1 is overexpressed in 62.6% of the ATC tissue specimens. These results indicated that the expression levels of RBX1 protein were significantly enhanced in ATC tissues (Fig. 1E). Immunoblotting indicated that the expression of RBX1 in the ATC cell lines was markedly higher compared with that in the PTC and thyroid cell lines (Fig. 1F, G). Next, the correlation analysis between RBX1 protein overexpression and ATC clinicopathological parameters indicated that the patients with low levels of RBX1 had a significantly longer overall survival time than those with high levels of RBX1 (Fig. 1H). Overall, these findings revealed the hypothesis that RBX1 plays a role in ATC progression and can be a potential biomarker for ATC diagnosis.

### RBX1 regulates the migration and invasiveness of ATC cells in vivo and in vitro

**Fig. 2:**
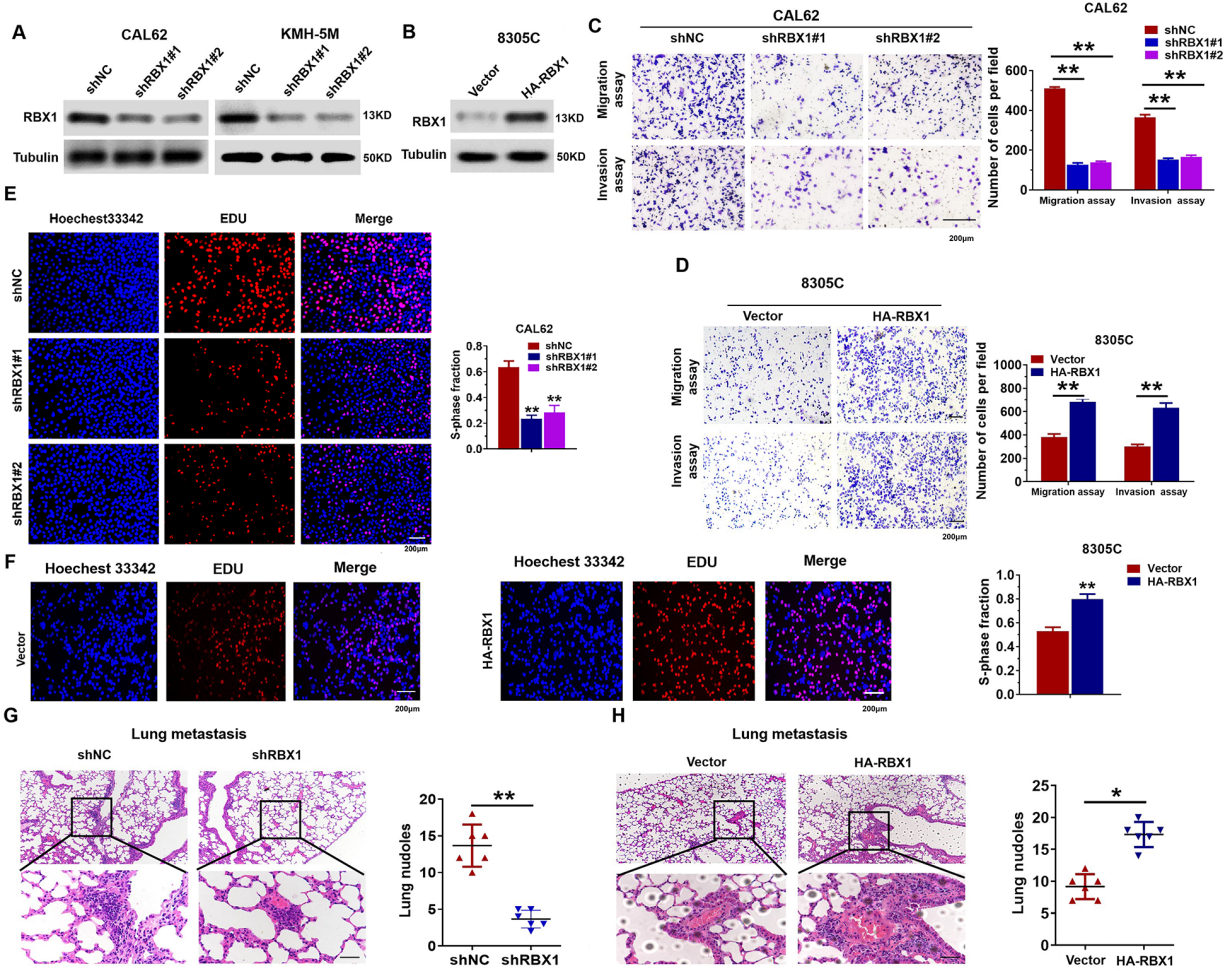
RBX1 facilitated the proliferation and migration of ATC cells. **A** Western blotting was performed to identify the RBX1 expression levels in CAL62 and KMH-5M cells stably transfected with shRBX1 plasmid. **B** RBX1 expression levels in the 8305C cells stably transfected with HA-RBX1 plasmid were measured using a western blot. **C** Transwell assays of CAL62 cells transfected with shRBX1 plasmid. **P < 0.01. **D** Transwell assays of 8305C cells transfected with HA-RBX1 plasmid. **P < 0.01. **E** EdU assays of CAL62 cells transfected with shRBX1 plasmid. **P < 0.01. **F** EdU assays of 8305C cells transfected with HA-RBX1 plasmid. **P < 0.01. **G**, **H** H&E staining of the sections of metastatic nodules in the lungs embedded with paraffin. *P < 0.05, **P < 0.01

We knocked down *RBX1* with subtype-specific shRNAs in two highly invasive ATC cell lines, namely, CAL62 and KMH-5M. Compared with scrambled shRNA, both shRBX1 and shRBX1 significantly reduced RBX1 expression in stable cell lines (Fig. 2A and Additional file 1: Fig. S1A, B) together with the stable overexpression of RBX1 in 8305C cell lines (Fig. 2B and Additional file 1: Fig. S1C). Transwell assays also revealed that the knockdown of RBX1 markedly inhibited the metastatic ability of KMH-5M and CAL62 cells, while the overexpression of RBX1 enhanced the metastatic ability of 8305C cells (Fig. 2C, D and Additional file 1: Fig. S1D-E). The proliferative capacity of the ATC cells was significantly suppressed by the knockdown of RBX1 in contrast to control cells. However, the overexpression of RBX1 significantly promoted their proliferative capacity (Fig. 2E, F and Additional file 2: Fig. S2). We conducted an in-depth investigation to understand the effects of RBX1 on the metastasis of ATC by constructing the tumor models in nude mice, which were classified into shRBX1 and shNC groups. Consecutive lung sections showed that the number of lung micrometastases in ATC patients was remarkably attenuated in the shRBX1 group (Fig. 2G). On the contrary, RBX1 overexpression enhanced the number of metastatic nodules in the lungs (Fig. 2H). In conclusion, these findings reflect that the stable RBX1 knockdown can suppress the metastasis together with the invasion of ATC in vivo and in vitro, and serve as a candidate oncogene in the development and metastasis of ATC.

### RBX1 promotes ATC development by increasing the Warburg effect

**Fig. 3:**
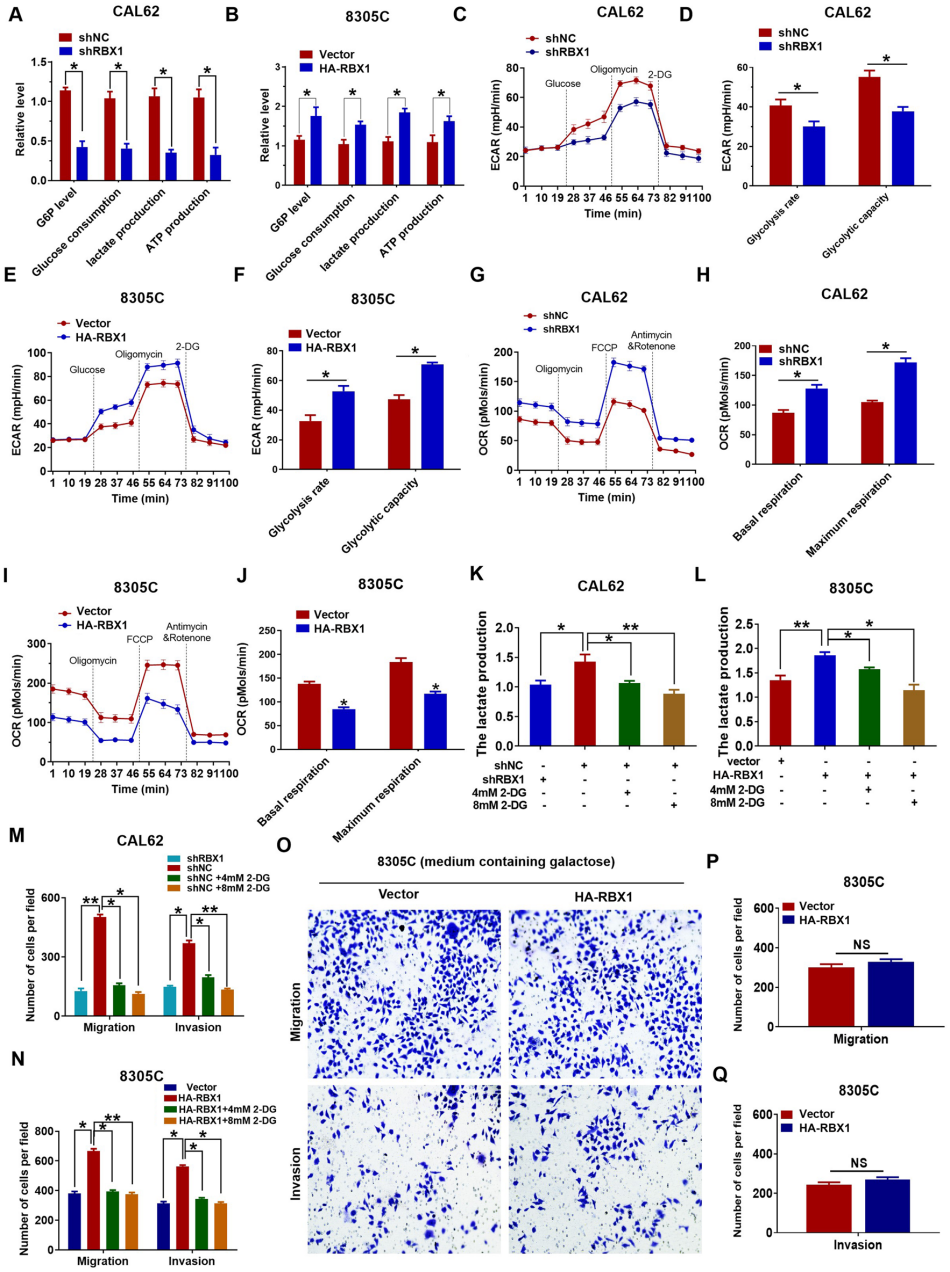
RBX1 promotes ATC progression by enhancing the Warburg effect. **A** Cellular glucose consumption, G6P levels, ATP levels, and lactate generation in CAL62/shRBX1 cells. *P < 0.05. **B** Cellular glucose consumption, G6P levels, ATP levels, and lactate generation in 8305C/HA-RBX1 cells. *P < 0.05. **C**, **D** ECAR data showing the glycolytic rate and capacity in CAL62/shRBX1 cells. **E**, **F** ECAR data showing the glycolytic rate and capacity in 8305C/HA-RBX1 cells. **G**, **H** OCR results showing basal respiration and maximum respiration in CAL62/shRBX1 cells. **I**, **J** OCR results showing basal respiration and maximum respiration in 8305C/HA-RBX1 cells. **K**, **L** Lactate generation by 8305C/p-RBCK1 or CAL62/shRBX1 cells in the presence of 2-DG. *P < 0.05, **P < 0.01. **M**, **N** Role of 2-DG in the invasion and migration of 8305C/p-RBCK1 or CAL62/shRBX1 cells. *P < 0.05, **P < 0.01. **O**–**Q** Culturing 8305C cells in a medium with galactose but without glucose abolished the impact of RBX1 overexpression on cell invasion and migration, NS = No Significant

E3 ubiquitin ligases can promote metabolic reprogramming in the development of different types of cancer [32, 33]. As the Warburg effect is characterized by a metabolic shift that is ubiquitous in the tumor cells, involving ATC, we investigated the effect of RBX1 on the glucose metabolism in ATC. Glucose-6-phosphate (G6P) levels, lactate generation, glucose consumption, and cellular levels of ATP were substantially reduced in CAL62 cells after RBX1 knockdown (Fig. 3A), whereas the overexpression of RBX1 led to the contrasting effect in 8305C cells (Fig. 3B). To further confirm the effect of RBX1 on ATC glycolysis, ECAR was determined, which indicates the overall glycolytic flux. The knockdown of RBX1 significantly attenuated the capacity and rate of glycolysis in the CAL62 cells (Fig. 3C, D), while the overexpression of RBX1 enhanced ECAR in the 8305C cells (Fig. 3E, F). As an indicator of mitochondrial respiration, OCR was enhanced in the CAL62/shRBX1 cells (Fig. 3G, H), whereas RBX1 overexpression decreased OCR in 8305C cells (Fig. 3I, J).

To investigate whether the Warburg effect led to the ATC cell development, 8305C/HA-RBX1 and CAL62/shRBX1 cells were treated with different concentrations of 2-deoxyglucose (2-DG) for one day. 2-DG remarkedly suppressed the glycolysis in 8305C/HA-RBX1 and CAL62/shRBX1 cells in a dose-dependent mode (Fig. 3K, L). Additionally, the invasive and migration ability of 8305C/HA-RBX1 and CAL62/shRBX1 cells were attenuated in a dose-dependent manner (Fig. 3M, N). To confirm that glycolysis regulates the invasion and migration of ATC, cells were cultivated in a medium containing galactose rather than glucose, thereby decreasing glycolytic flux and driving them to be dependent on oxidative phosphorylation. This significantly decreased the increase in 8305C cell invasion and migration resulting from the overexpression of RBX1 (Fig. 3O–Q). These results display that RBX1 inhibits oxidative phosphorylation while facilitating the aerobic glycolysis in ATC cells, but facilitates invasion and migration by enhancing the Warburg effect in the ATC cell lines.

### PKM2 is essential for RBX1 to enhance the Warburg effect

**Fig. 4:**
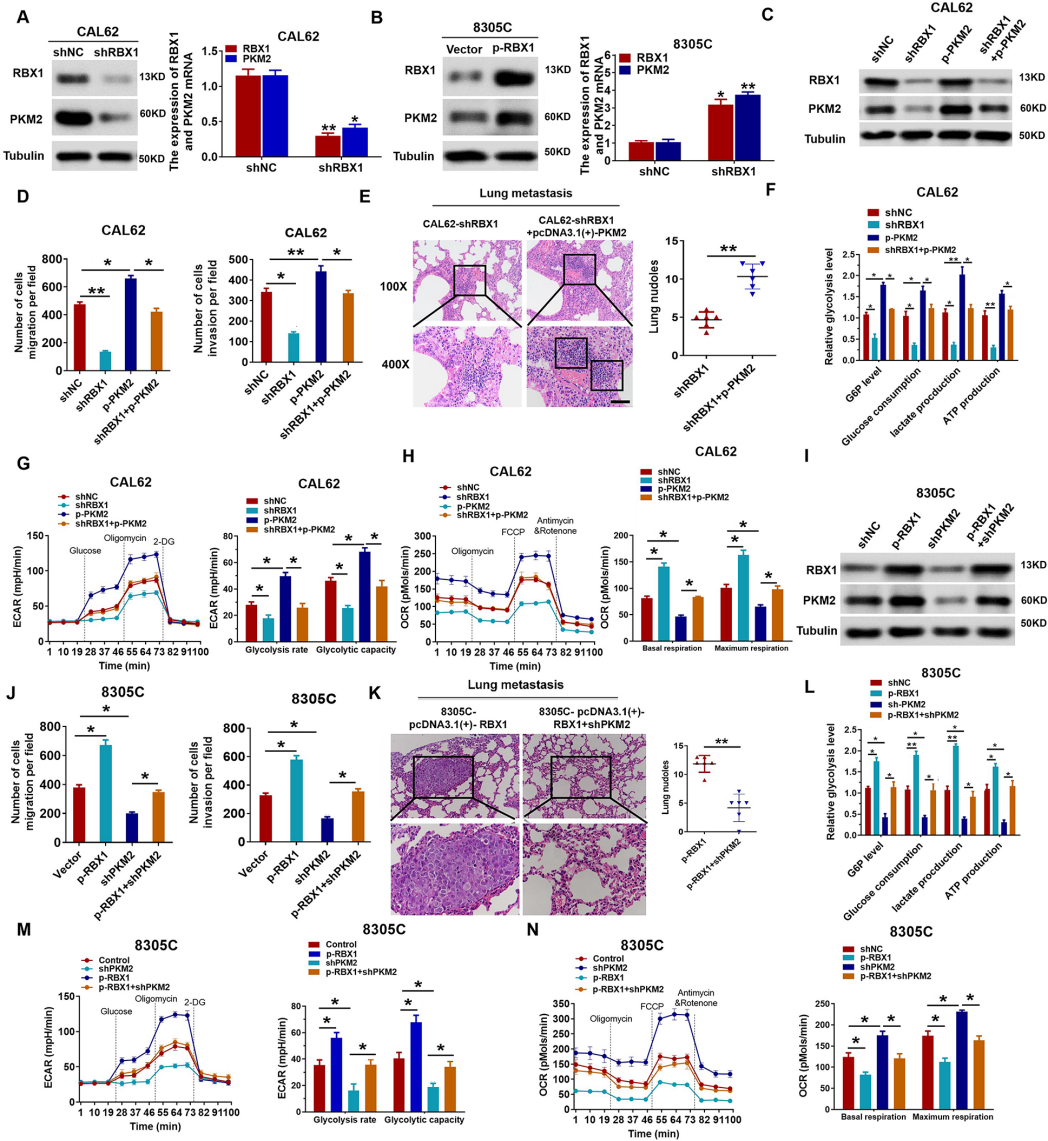
RBX1 promotes ATC progression by upregulating PKM2 expression. **A,** **B** qRT-PCR and western blotting were performed for measuring the PKM2 and RBX1 expression. **C** Western blotting was performed to determine the PKM2 and RBX1 expression in various groups. **D** Quantification of transwell assay in various groups. *P < 0.05, **P < 0.01. **E** Quantification and representative images of the lung metastases in various groups of nude mice (n = 6). **F** Cellular glucose consumption, G6P levels, ATP levels, and lactate generation in the specific groups. *P < 0.05, **P < 0.01. **G**, **H** Measurement of OCR and ECAR in the specific groups. *P < 0.05. **I** PKM2 and RBX1 expression levels in various groups were determined using western blotting. J. Quantification of transwell assay in diverse groups. *P < 0.05, **P < 0.01. **K** Quantification and representative images of the lung metastases in various groups of nude mice (n = 6). **L** Cellular glucose consumption, G6P levels, ATP levels, and lactate generation in the specific groups. *P < 0.05, **P < 0.01. **M**, **N** ECAR and OCR were measured in the indicated groups. *P < 0.05, **P < 0.01

In contrast to PKM1, higher PKM2 expression is a critical factor for promoting the Warburg effect in cancer cells compared to normal cells. Decreased expression of PKM2 might result in the suppression of the Warburg effect, thereby suppressing the tumorigenic potential [34, 35]. Thus, we determined whether RBX1 regulates PKM2 expression by initially measuring the PKM2 expression in RBX1-knockdown and -overexpressing ATC cells. Western blotting analysis revealed that the knockdown of RBX1 significantly reduced the expression of PKM2 in CAL62 cells (Fig. 4A). In contrast, the RBX1 overexpression markedly enhanced the expression of PKM2 in 8305C cells (Fig. 4B). Additionally, the enhanced PKM2 levels reversed the reduction of PKM2 expression in CAL62/shRBX1 cells (Fig. 4C). Rescue experiments indicated that the restoration of PKM2 expression could abrogate the decrease in metastatic capacity of ATC resulting from *RBX1* silencing (Fig. 4D). Figure 4F shows in vivo tumor metastasis, and the ATP levels in ATC cells. Simultaneously, the knockdown of RBX1 attenuated ECAR in ATC cells, while concomitant PKM2 overexpression decreased the reduction in glycolytic capacity and rate (Fig. 4G, H).

Subsequently, we discussed the effect of attenuated PKM2 expression on the PKM2 and RBX1 protein levels as well as on the cell invasion and migration in 8305C cells with RBX1 overexpression. Western blotting results demonstrated that the overexpression of RBX1 significantly enhanced PKM2 expression, while the knockdown of PKM2 largely attenuated the increase in PKM2 expression caused by RBX1 in 8305C cells (Fig. 4I). At the same time, a reduction in PKM2 significantly decreased the cell invasion and migration enhanced by RBX1 (Fig. 4J). Besides, the analysis of in vivo metastasis revealed that the decrease in PKM2 reduced the incidence of pulmonary and intrahepatic metastasis in the 8305C-RBX1 group (Fig. 4K). PKM2 knockdown led to a reduction in G6PD activity, ATP levels, lactate generation, and glucose consumption mediated by RBX1 in ATC cells (Fig. 4L). The overexpression of RBX1 upregulated ECAR in ATC cells, while the concomitant PKM2 knockdown hindered the increase in glycolytic capacity and rate (Fig. 4M, N). These results display that PKM2 is a type of functional downstream target of RBX1 to modulate aerobic glycolysis and is essential for *RBX1*-mediated tumor development.

### RBX1 regulates *PKM* alternative splicing to promote the PKM2-mediated Warburg effect

**Fig. 5:**
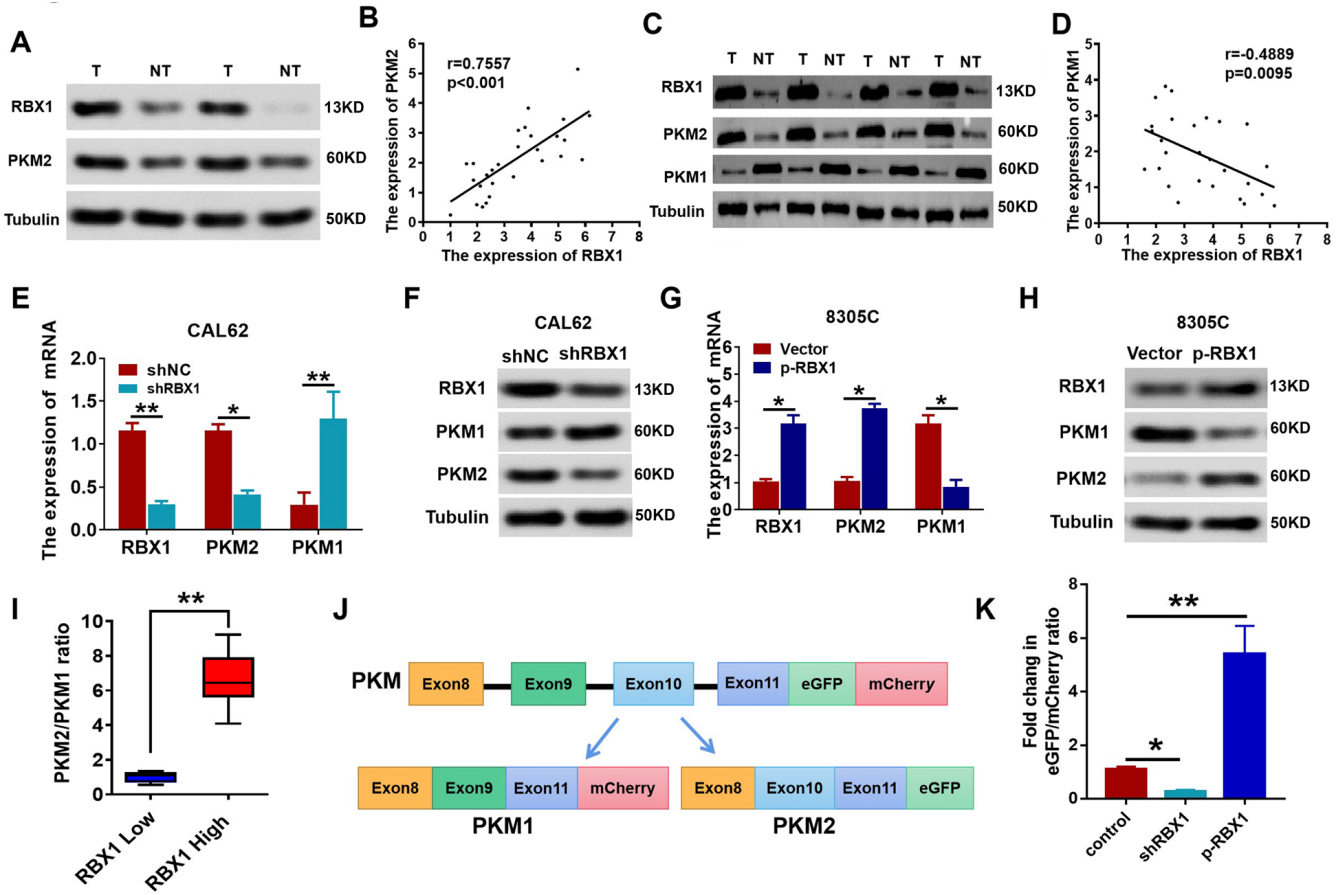
RBX1 regulates *PKM* alternative splicing. **A** Western blot was performed to determine the PKM2 and RBX1 protein expression levels in ATC tissues together with the normal tissues adjacent to the tumor. **B** Scatter plots of RBX1 and PKM2 protein expression in ATC. **C** Western blot was performed to determine the PKM1 and RBX1 protein expression levels in ATC tissues together with the normal tissues adjacent to the tumor. **D** Scatter plots of RBX1 and PKM1 protein expression in ATC. **E** qRT-PCR showing expression levels of PKM1 and PKM2 in shRBX1-CAL62 cells. **F** Western blotting showing expression levels of PKM1 and PKM2 in shRBX1-CAL62 cells. **G** qRT-PCR showing expression levels of PKM1 and PKM2 in HA-RBX1 8305C cells. **H** Western blotting showing expression levels of PKM1 and PKM2 in HA-RBX1 8305C cells. **I** Compared to the tissue samples with low RBX1 expression, the PKM2/PKM1 ratio was significantly higher in those with high RBX1 expression. **J**, **K** Relative fluorescence intensities of mCherry and eGFP were quantified using ImageJ, and the fold change in the eGFP/mCherry ratio was calculated for the HA-RBX1, shRBX1, and control groups

Cancer cells achieve the metabolic advantage over normal cells by modulating *PKM* alternative splicing and facilitating the PKM2 expression [19]. To further demonstrate whether RBX1 is responsible for this phenomenon, we detected the expression levels of RBX1, PKM2, and PKM1 in ATC patient samples and then compared these levels with those in the corresponding adjacent non-cancerous tissues using western blotting. PKM2 and RBX1 protein levels were significantly enhanced in ATC tissues. Besides, scatter plots exhibited a positive association between the expression of PKM2 and RBX1 (Fig. 5A, B). However, the expression of PKM1 was markedly low in the ATC tissues, and the expression of RBX1 and PKM1 were negatively correlated (Fig. 5C, D). Based on the positive relationship between the expression of PKM2 and RBX1, together with the inverse relationship between PKM1 and RBX1 observed in ATC, these findings display that RBX1 exerts a crucial role in modulating the alternative splicing of *PKM*.

To investigate the effect of RBX1 on modulating *PKM* alternative splicing, the expression of *PKM* isoform was identified in CAL62 cells after the depletion of shRNA-mediated RBX1 expression by quantitative western blotting and qRT-PCR. At the level of mRNA, *RBX1* knockdown in CAL62 cells led to enhanced *PKM1* and attenuated expression of *PKM2* (Fig. 5E). An in-depth analysis of the expression of *PKM* isoforms at the protein level in cells with RBX1 depletion indicated an increase in PKM1 and a decrease in PKM2, which was in accordance with the RNA expression data (Fig. 5F). Furthermore, both western blotting and qRT-PCR demonstrated that the increase in RBX1 significantly enhanced the PKM2 expression and attenuated the PKM1 expression in 8305C cells (Fig. 5G, H). Next, we analyzed the expression of PKM1 and PKM2 isoforms in the tissue samples with low or high RBX1 expression. The PKM2/PKM1 ratio was significantly higher in the tissue samples with high expression of RBX1 when compared with those with low expression of RBX1 (Fig. 5I). To further demonstrate the effect of RBX1 on the modulation of *PKM* alternative splicing, we produced a dual reporter PKM minigene system. The eGFP/mCherry ratio with the overexpression of RBX1 increased 5.4-fold compared with that in the control. RBX1 knockdown led to a 3.2-fold decrease in the eGFP/mCherry ratio compared with that in the control, which elucidated its effect on modulating the *PKM* alternative splicing (Fig. 5J, K). Together, these studies confirmed that RBX1 regulates *PKM* alternative splicing to promote the PKM2-mediated Warburg effect in ATC cells.

### RBX1 regulates *PKM* alternative splicing via SMAR1/HDAC6 complex degradation

**Fig. 6:**
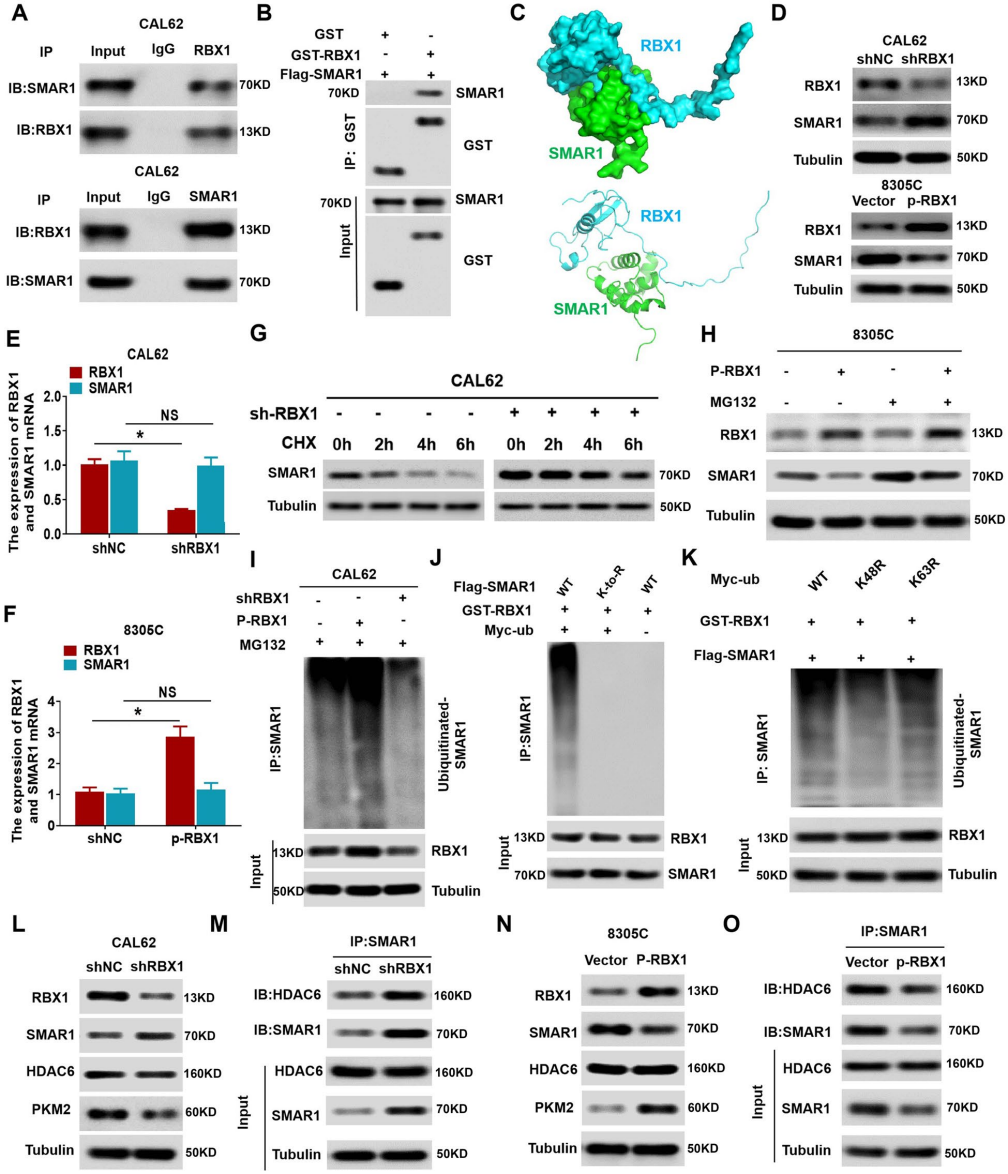
RBX1 regulates *PKM* alternative splicing via SMAR1/HDAC6 complex degradation. **A** Co-IP showing direct binding of endogenous RBX1 and SMAR1 in CAL62 cells. **B** GST pull-down assay showing direct binding of endogenous RBX1 and SMAR1. **C** Top-ranked docking confirmation. 3D structures of SMAR1 and RBX1, with SMAR1 and RBX1 shown in green and cyan. **D** Western blotting was performed to detect the expression of RBX1 and SMAR1 in different groups. **E**, **F** qRT-PCR was performed to detect the expression of RBX1 and SMAR1 in different groups. G. CAL62 cells were transfected with shRBX1 plasmid. After that, cells were exposed to 20 μmol/L cyclohexanone (CHX) at the given times, and the SMAR1 degradation was identified using western blotting. **H** 8305C cells were treated with 10 μM MG132, while the RBX1 expression was altered. The SMAR1 protein expression level was determined by western blotting. **I** CAL62 cells were treated with 10 μM MG132 while transfecting them with HA-RBX1 or shRBX1 plasmids. Subsequently, the level of ubiquitin bound to the SMAR1 protein was measured by Co-IP. **J** Wild-type SMAR1 or K- to -R mutations in ATC cells (mutations in all the Lys positions of SMAR1 gene) for ubiquitination. **K** Determination of the type of SMAR1 ubiquitination in ATC cells. **L** Western blotting showing expression levels of RBX1, SMAR1, HDAC6, and PKM2 in shRBX1-CAL62 cells. **M** Co-IP combined with western blotting showing the expression levels of SMAR1 and HDAC6 in shRBX1-CAL62 cells. **N** Western blotting showing expression levels of RBX1, SMAR1, HDAC6, and PKM2 in HA-RBX1-8305C cells. **O** Co-IP combined with western blotting showing the expression levels of SMAR1 and HDAC6 in HA-RBX1-8305C cells

We initially attempted to identify RBX1-interacting proteins in ATC cells using mass spectrometry. As shown in Additional file 5: Table S1, we identified several reported RBX1-interacting proteins, including TLE3, FoxA1, and SHP-1, as well as a previously unreported RBX1 interactor, namely, SMAR1. These experiments validated the direct interaction of HDAC6 with SMAR1 and the coexistence of SMAR1-HDAC6 as a complex. The generation of such a complex demonstrates the molecular dynamics between the proteins in modulating SMAR1-mediated *PKM* alternative splicing. Therefore, we hypothesized that RBX1 regulates *PKM* alternative splicing via the degradation of the SMAR1/HDAC6 complex. Co-IP analysis revealed an interaction between SMAR1 and RBX1 (Fig. 6A and Additional file 3: Fig. S3A-B). We observed that purified GST-RBX1 was bound to FLAG-tagged SMAR1 in vitro (Fig. 6B). Docking analysis suggested binding interactions between SMAR1 and RBX1 (Fig. 6C). These findings indicated that RBX1 binds with SMAR1 in ATC cells. Further analysis of PKM isoform expression in shSMAR1 cells at the protein level indicated reduced expression of PKM1 and upregulation of PKM2 (Additional file 4: Fig. S4). Moreover, as shown in Fig. 6D, RBX1 knockdown upregulated the level of SMAR1 protein in CAL62 cells, whereas RBX1 overexpression significantly reduced SMAR1 expression in 8305C cells. However, the expression of *SMAR1* mRNA was not influenced by RBX1 alteration in the ATC cells (Fig. 6E, F), indicating that RBX1 regulated the expression of SMAR1 after it has been translated.

Previous studies have proven that UPS regulated the polyubiquitination and degradation of SMAR1 [19]. As the E3 ubiquitin ligase, RBX1, plays a role in protein degradation and recycling, we initially determined the degeneration of SMAR1 protein in RBX1-knockdown cells after inhibition of protein synthesis using cycloheximide (CHX). As shown in Fig. 6G, silencing *RBX1* significantly inhibited SMAR1 degradation in ATC cells. Moreover, we observed that the levels of SMAR1 protein were restored in the cells with RBX1 overexpression after treatment with the proteasome inhibitor MG132 (Fig. 6H). Second, RBX1 ectopic expression led to an enhanced SMAR1 ubiquitination, while the RBX1 knockdown reduced SMAR1 polyubiquitination (Fig. 6I). Eventually, these findings revealed that the mutations in all the Lys positions of SMAR1 abolished the SMAR1 polyubiquitination induced by RBX1 in ATC cells (Fig. 6J). Furthermore, the mutation of Lys48 position on the ubiquitin nearly abolished the RBX1-mediated ubiquitination of SMAR1, while the mutation of K63R on ubiquitin did not exhibit any effect (Fig. 6K). These findings revealed that RBX1 is responsible for SMAR1 degradation via the ubiquitin–proteasome pathway in ATC.

Finally, we determined whether RBX1 influenced *PKM* alternative splicing via the destruction of the SMAR1/HDAC6 complex. The changes in the expression of HDAC6, SMAR1, PKM2, and PKM1 as well as the changes in the SMAR1/HDAC6 complex, were determined in CAL62 cells with *RBX1* knockdown. It can be observed that *RBX1* knockdown in CAL62 cells significantly increased the levels of SMAR1 expression, SMAR1/HDAC6 complex, and PKM1 and decreased the expression of PKM2. However, no variations were observed in the HDAC6 protein levels (Fig. 6L, M). In contrast, the RBX1 overexpression in the 8305C cells significantly attenuated the expression of SMAR1, the SMAR1/HDAC6 complex, and PKM1, and increased PKM2 expression (Fig. 6N, O). Together, these results indicated that ATC cell aerobic glycolysis and migration resulting from the modulation of RBX1-mediated *PKM* alternative splicing depends on the destruction of the SMAR1/HDAC6 complex.

### Oncogenic effect of RBX1 is dependent on SMAR1 destabilization

**Fig. 7:**
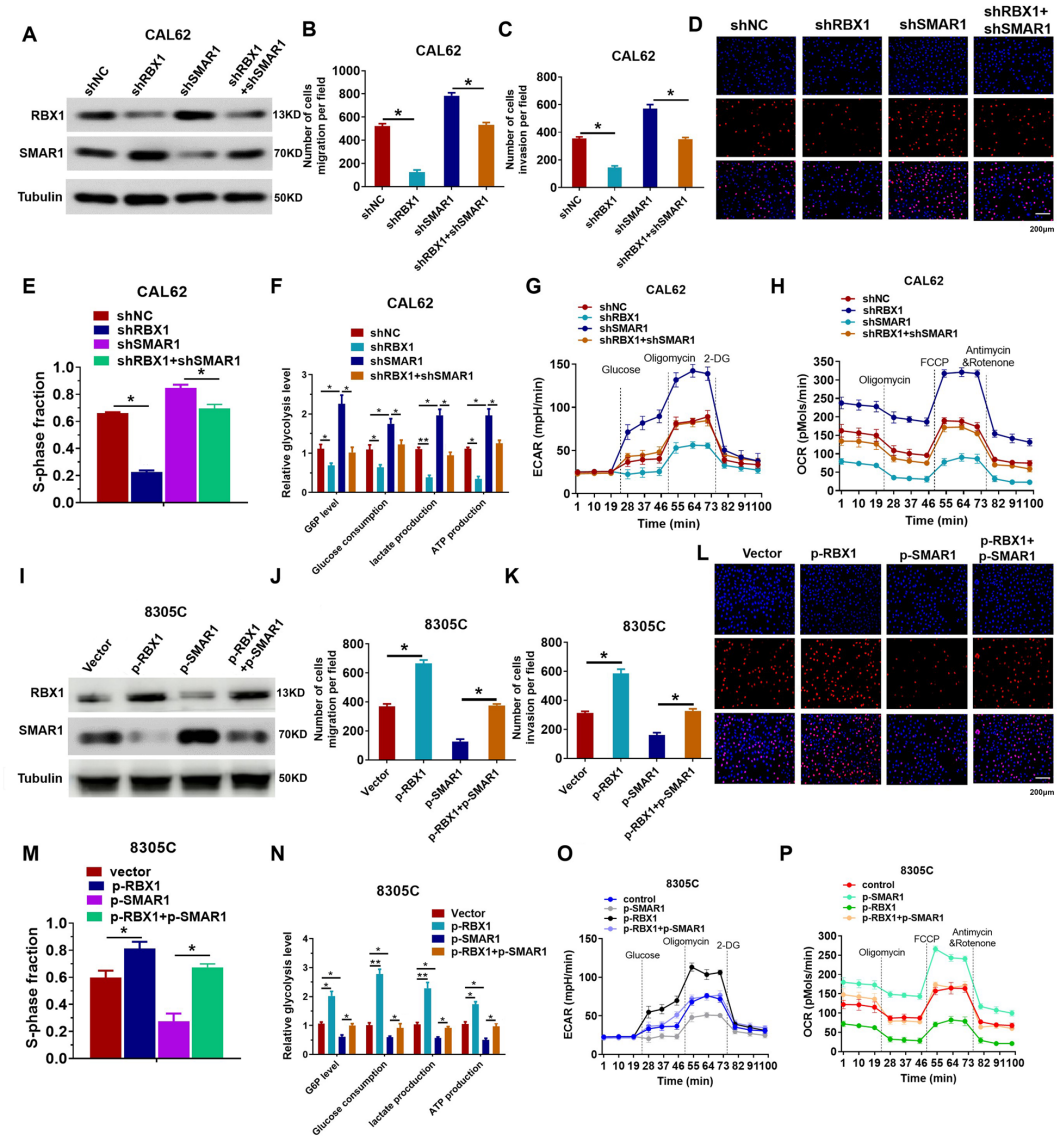
RBX1 promotes ATC migration and aerobic glycolysis via SMAR1. **A** Western blotting was performed to determine the SMAR1 and RBX1 expression in various groups. **B**, **C** Quantification of transwell assay in various groups. *P < 0.05, **P < 0.01. **D**, **E** Quantification of EdU assays in different groups. *P < 0.05, **P < 0.01. **F** Cellular glucose consumption, G6P levels, ATP levels, and lactate generation in specific groups. *P < 0.05, **P < 0.01. **G**, **H** Measurement of OCR and ECAR in specific groups. *P < 0.05. **I** SMAR1 and RBX1 expression levels in various groups were determined using western blotting. **J**, **K** Quantification of transwell assays in different groups. *P < 0.05, **P < 0.01. **L**, **M** Quantification of EdU assays in different groups. *P < 0.05, **P < 0.01. **N** Cellular glucose consumption, G6P levels, ATP levels, and lactate generation in specific groups. *P < 0.05, **P < 0.01. **O**, **P** ECAR and OCR were measured in the indicated groups. *P < 0.05, **P < 0.01

We transfected the shSMAR1 plasmid into *RBX1*-knockdown ATC cells and measured its effects on biological function. *RBX1* knockdown significantly increased SMAR1 protein expression, whereas the *SMAR1* knockdown attenuated the increased expression of SMAR1 after *RBX1* knockdown in ATC cells (Fig. 7A). Rescue tests indicated that the reduced expression of SMAR1 abrogated the RBX silencing-induced reduction in the metastatic capacity of ATC cells (Fig. 7B, C). A reduction in SMAR1 resulted in a rescue of the attenuated proliferation capacity of CAL62/shRBX1 cells (Fig. 7D, E). Knockdown of SMAR1 rescued reduction in glucose consumption, G6P, ATP levels, and lactate generation mediated by shRBX1 in ATC cells (Fig. 7F). Simultaneously, shRBX1 reduced ECAR in ATC cells, while SMAR1 knockdown decreased the reduction in glycolytic capacity and rate (Fig. 7G, H). In contrast, SMAR1 upregulation largely decreased the RBX1 increased cell invasion and migration (Fig. 7I, K), thereby decreasing the proliferation capacity of the 8305C/RBX1 group (Fig. 7L, M). Furthermore, SMAR1 upregulation rescued the increase in glucose consumption, G6P, ATP levels, and RBX1-mediated lactate generation in ATC cells (Fig. 7N). The overexpression of RBX1 enhanced ECAR in ATC cells, while the simultaneous increase in SMAR1 led to a decrease in the enhanced glycolytic capacity and rate (Fig. 7O, P). In conclusion, these results demonstrated that RBX1 facilitates the migration and aerobic glycolysis in ATC through SMAR1.

## Discussion

ATC is one of the most aggressive human cancers with a rapid progression and poor prognosis. After the diagnosis of ATC, the average survival time is below 6 months, and the 5-year survival rate ranged from 0 to 10% [36]. Till now, malignant metastasis is the primary reason for treatment failure in ATC patients. Therefore, it is extremely important to better understand the biological features of ATC. In the past several years, the significance of metabolic reprogramming in tumorigenesis and its therapeutic implications have been demonstrated. Metabolic reprogramming is regarded as a hallmark of cancer. ATC is a deadly disease as the tumors display metabolic variability and the survival rate of ATC patients is very low [36]. As a result, understanding the molecular basis of ATC from a metabolic perspective is important for better disease management. This study indicated that RBX1 has a high expression in the ATC tissues and enhanced RBX1 expression is associated with survival and malignancy in ATC patients. Moreover, it highlighted that RBX1 exerts a critical effect on the reprogramming of glucose metabolism in ATC.

RBX1 is a critical component of the E3 ubiquitin ligase [30]. Considering its evolution, it is conserved in both yeast and humans and exerts a significant role in embryonic development. In recent years, it has been shown that RBX1 exerts a major role in the modulation of several cellular physiological functions and is simultaneously associated with carcinogenesis [30]. In several primary tumors, including lung cancer, non-muscle invasive bladder transitional cell carcinoma (NMIBC), and liver cancer, RBX1 is overexpressed. Some studies have revealed that this enhanced expression is significantly correlated with malignant characteristics, including deeper infiltration, lymph-node metastasis, lymphatic infiltration, and venous infiltration [24, 37]. However, no information is available on the detailed molecular mechanisms and the role of RBX1 in ATC. In this study, our data reflected that the expression of RBX1 was higher in tumors of ATC patients when compared with that in the relevant non-tumor tissues. High levels of RBX1 were strongly related to tumor metastasis and short overall survival in ATC patients. RBX1 suppressed oxidative phosphorylation while facilitating aerobic glycolysis in ATC cells. However, it facilitated invasion and migration by enhancing the Warburg effect in ATC cells. Therefore, these results are significant to better understand the biological functions of RBX1 in ATC and evaluate the potential of RBX1 as a therapeutic target.

Cancer cells exhibit a metabolic advantage by modulating alternative splicing of *PKM* to facilitate PKM2 expression. Therefore, therapeutic targeting of this splicing has proven to be an effective strategy for cancer treatment. For example, Calabretta et al. reported that the modulation of the *PKM* alternative splicing in the PDAC cells confers drug resistance, resulting in the transition to drug-resistant PDAC (DR-PDAC) [17]. Yuki Kuranaga et al. demonstrated that *SRSF3* silencing induces significant growth inhibition in human colon cancer cells and leads to an enhanced PKM1/PKM2 ratio. This causes a metabolic transition from glycolysis to oxidative phosphorylation [37]. Therefore, the identification of mechanisms controlling *PKM* alternative splicing to promote the PKM2-mediated Warburg effect is important for overcoming the current challenges in ATC treatment. Herein, we described a novel mechanism for ATC treatment, involving RBX1-mediated degradation of the SMAR1/HDAC6 complex. First, we observed that PKM2 is a type of functional downstream target of RBX1 in modulating aerobic glycolysis, which is essential for RBX1-mediated tumor progression. Furthermore, our data demonstrated that RBX1 regulates alternative splicing to promote the PKM2-mediated Warburg effect in ATC cells. Moreover, ATC cell migration and aerobic glycolysis induced by regulating RBX1-mediated *PKM* alternative splicing are determined by the degradation of the SMAR1/HDAC6 complex. Overall, these results demonstrated that RBX1 controls the degradation of the SMAR1/HDAC6 complex to affect ATC progression, which helps in the development of a novel regulatory mechanism for *PKM* alternative splicing.

Finally, we studied the degradation of the SMAR1/HDAC6 complex mediated by RBX1. Post-translational modification of E3 ubiquitin ligase can regulate the function, fate, and intracellular mechanism of the target proteins [38]. RBX1 interacts with multiple substrates to play a key role [30, 39]. Ubiquitin proteasome-mediated SMAR1 degradation is the key mechanism that regulates SMAR1 levels in cells. Our findings suggested that RBX1 disrupts the SMAR1/HDAC6 complex by facilitating the degradation and ubiquitination of SMAR1. This was highlighted based on the following observations. First, RBX1 binds directly with SMAR1 in ATC cells. Second, the downregulation of RBX1 significantly reduced the ubiquitination of SMAR1, while overexpression of RBX1 promoted the ubiquitination of SMAR1. Third, RBX1 can reduce the half-life of SMAR1. Finally, our data revealed that all the Lys site mutations of SMAR1 can eliminate RBX1-induced SMAR1 polyubiquitination in ATC cells. Lys48 mutation of ubiquitin almost eliminated the RBX1-mediated SMAR1 ubiquitination, while the K63R mutation on the ubiquitin had no impact.

**Fig. 8:**
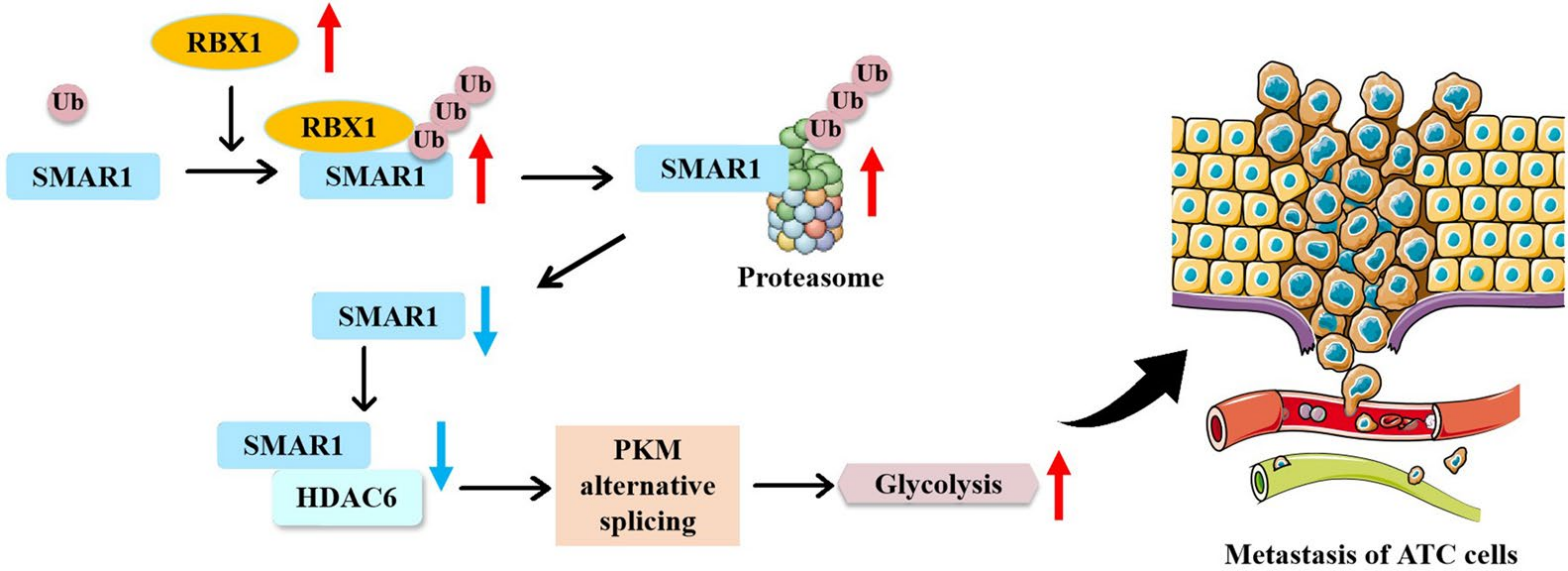
Proposed model for the E3 ubiquitin ligase RBX1-mediated regulation *PKM* alternative splicing to facilitate anaplastic thyroid carcinoma metastasis and aerobic glycolysis by destroying the SMAR1/HDAC6 complex

Taken together, this study provides the first evidence that RBX1 is highly associated with ATC progression and should be considered as a biomarker for ATC diagnosis. Additionally, we revealed that RBX1 inhibits oxidative phosphorylation while facilitating aerobic glycolysis in ATC cells, simultaneously facilitating invasion and migration by enhancing the Warburg effect in ATC cell lines. PKM2 is a type of functional downstream target of RBX1 in modulating aerobic glycolysis, which is essential for RBX1-mediated tumor progression. In summary, our findings demonstrated that RBX1 regulates *PKM* alternative splicing to promote the PKM2-mediated Warburg effect by destroying the SMAR1/HDAC6 complex in ATC cells. Moreover, RBX1 regulates SMAR1 expression by direct RBX1-SMAR1 binding and facilitating its degradation and ubiquitination (Fig. 8). Based on these results, RBX1 should be considered as the candidate biomarker for diagnosis and treatment of ATC.

## Supplementary Information

**Additional file 1: Figure S1. **RBX1 promoted the migration of ATC cells. A-B. qRT-PCR was used to detect the expression levels of RBX1 in CAL62 and KMH-5M cells stably transfected with the shRBX1 plasmid. C. qRT-PCR was used to detect the expression levels of RBX1 in 8305C cells stably transfected with the HA-RBX1 plasmid. D-E. Transwell assays of KMH-5M cells transfected with shRBX1 plasmid. **P < 0.01.

**Additional file 2: Figure S2. **RBX1 promoted the proliferation of ATC cells. A, B. Edu assays of KMH-5M cells transfected with shRBX1 plasmid. **P < 0.01. C-D. CCK8 assays of CAL62 and 8305 cells transfected with shRBX1 plasmid or HA-RBX1 plasmid.

**Additional file 3: Figure S3. **Co-IP showing direct binding of endogenous RBX1 and SMAR1. A-B.Co-IP showing direct binding of endogenous RBX1 and SMAR1 in 8305C cells.

**Additional file 4: Figure S4. **Expression of PKM isoforms upon shRNA-mediated knockdown of SMAR1 in 8305C by western blot.

**Additional file 5: Table S1.** RBX1-interacting proteins in ATC cells using mass spectrometry.

## Data Availability

All the data produced or analyzed in this study are included in the published article. The additional datasets analyzed and/or used in this study are available from the relevant authors upon reasonable request.

## Abbreviations

PTC: Papillary thyroid cancer
ATC: Anaplastic thyroid cancer
PKL: Pyruvate kinase liver
PKR: Pyruvate kinase
RBC: Red blood cell
H&E: Haematoxylin and eosin
IHC: Immunohistochemical
shRNA: ShRNA-mediated double stranded RNA
OCR: Oxygen consumption rate
ECAR: Extracellular acidification rate
Co-IP: Co-immunoprecipitation
OS: Overall survival
G6P: Glucose-6-phosphate
DR-PDAC: Drug-resistant PDAC
NMIBC: Non-muscle invasive bladder transitional cell carcinoma
2-DG: 2-Deoxyglucose
UPS: Ubiquitin proteasome system
SCF: Skp1/Cullin/F-box

## References

[1] Saini S, Tulla K, Maker AV, Burman KD, Prabhakar BS. Therapeutic advances in anaplastic thyroid cancer: a current perspective. Mol Cancer. 2018;17(1):154. 30352606 10.1186/s12943-018-0903-0 PMC6198524

[2] Coca-Pelaz A, Shah JP, Hernandez-Prera JC, Ghossein RA, Rodrigo JP, Hartl DM, et al. Papillary thyroid cancer-aggressive variants and impact on management: a narrative review. Adv Ther. 2020;37(7):3112–28. 32488657 10.1007/s12325-020-01391-1 PMC7467416

[3] Lu YL, Huang YT, Wu MH, Chou TC, Wong RJ, Lin SF. Efficacy of adavosertib therapy against anaplastic thyroid cancer. Endocr Relat Cancer. 2021;28(5):311–24. 33769310 10.1530/ERC-21-0001 PMC8197631

[4] Pozdeyev N, Rose MM, Bowles DW, Schweppe RE. Molecular therapeutics for anaplastic thyroid cancer. Semin Cancer Biol. 2020;61:23–9. 31991166 10.1016/j.semcancer.2020.01.005 PMC7117889

[5] Hu S, Ma J, Su C, Chen Y, Shu Y, Qi Z, et al. Engineered exosome-like nanovesicles suppress tumor growth by reprogramming tumor microenvironment and promoting tumor ferroptosis. Acta Biomater. 2021;135:567–81. 34506976 10.1016/j.actbio.2021.09.003

[6] Schiliro C, Firestein BL. Mechanisms of metabolic reprogramming in cancer cells supporting enhanced growth and proliferation. Cells. 2021;10(5):1056. 33946927 10.3390/cells10051056 PMC8146072

[7] Orang AV, Petersen J, McKinnon RA, Michael MZ. Micromanaging aerobic respiration and glycolysis in cancer cells. Mol Metab. 2019;23:98–126. 30837197 10.1016/j.molmet.2019.01.014 PMC6479761

[8] Wu S, Cao R, Tao B, Wu P, Peng C, Gao H, et al. Pyruvate facilitates FACT-mediated gammaH2AX loading to chromatin and promotes the radiation resistance of glioblastoma. Adv Sci (Weinh). 2022;9(8): e2104055. 35048565 10.1002/advs.202104055 PMC8922107

[9] Tang D, Subramanian J, Haley B, Baker J, Luo L, Hsu W, et al. Pyruvate kinase muscle-1 expression appears to drive lactogenic behavior in CHO cell lines, triggering lower viability and productivity: a case study. Biotechnol J. 2019;14(4): e1800332. 30179303 10.1002/biot.201800332

[10] David CJ, Manley JL. Alternative pre-mRNA splicing regulation in cancer: pathways and programs unhinged. Genes Dev. 2010;24(21):2343–64. 21041405 10.1101/gad.1973010 PMC2964746

[11] Bonomi S, Gallo S, Catillo M, Pignataro D, Biamonti G, Ghigna C. Oncogenic alternative splicing switches: role in cancer progression and prospects for therapy. Int J Cell Biol. 2013;2013: 962038. 24285959 10.1155/2013/962038 PMC3826442

[12] Christofk HR, Vander Heiden MG, Harris MH, Ramanathan A, Gerszten RE, Wei R, et al. The M2 splice isoform of pyruvate kinase is important for cancer metabolism and tumour growth. Nature. 2008;452(7184):230–3. 18337823 10.1038/nature06734

[13] Yang W, Xia Y, Hawke D, Li X, Liang J, Xing D, et al. PKM2 phosphorylates histone H3 and promotes gene transcription and tumorigenesis. Cell. 2012;150(4):685–96. 22901803 10.1016/j.cell.2012.07.018 PMC3431020

[14] Li YH, Li XF, Liu JT, Wang H, Fan LL, Li J, et al. PKM2, a potential target for regulating cancer. Gene. 2018;668:48–53. 29775756 10.1016/j.gene.2018.05.038

[15] Huang Y, Chen LM, Xie JY, Han H, Zhu BF, Wang LJ, et al. High expression of PKM2 was associated with the poor prognosis of acute leukemia. Cancer Manag Res. 2021;13:7851–8. 34675679 10.2147/CMAR.S331076 PMC8520821

[16] Wang C, Jiang J, Ji J, Cai Q, Chen X, Yu Y, et al. PKM2 promotes cell migration and inhibits autophagy by mediating PI3K/AKT activation and contributes to the malignant development of gastric cancer. Sci Rep. 2017;7(1):2886. 28588255 10.1038/s41598-017-03031-1 PMC5460252

[17] Calabretta S, Bielli P, Passacantilli I, Pilozzi E, Fendrich V, Capurso G, et al. Modulation of PKM alternative splicing by PTBP1 promotes gemcitabine resistance in pancreatic cancer cells. Oncogene. 2016;35(16):2031–9. 26234680 10.1038/onc.2015.270 PMC4650269

[18] Zhu S, Chen W, Wang J, Qi L, Pan H, Feng Z, et al. SAM68 promotes tumorigenesis in lung adenocarcinoma by regulating metabolic conversion via PKM alternative splicing. Theranostics. 2021;11(7):3359–75. 33537092 10.7150/thno.51360 PMC7847678

[19] Choksi A, Parulekar A, Pant R, Shah VK, Nimma R, Firmal P, et al. Tumor suppressor SMAR1 regulates PKM alternative splicing by HDAC6-mediated deacetylation of PTBP1. Cancer Metab. 2021;9(1):16. 33863392 10.1186/s40170-021-00252-x PMC8052847

[20] Xie L, Dang Y, Xie J. Post-translational modification and regulatory network of Mycobacterium tuberculosis antibiotic resistance. Sheng Wu Gong Cheng Xue Bao. 2018;34(8):1279–87. 30152213 10.13345/j.cjb.170530

[21] Tripodi F, Nicastro R, Reghellin V, Coccetti P. Post-translational modifications on yeast carbon metabolism: regulatory mechanisms beyond transcriptional control. Biochim Biophys Acta. 2015;1850(4):620–7. 25512067 10.1016/j.bbagen.2014.12.010

[22] Hermand D. F-box proteins: more than baits for the SCF? Cell Div. 2006;1:30. 17166256 10.1186/1747-1028-1-30 PMC1712225

[23] Wei D, Sun Y. Small RING finger proteins RBX1 and RBX2 of SCF E3 ubiquitin ligases: the role in cancer and as cancer targets. Genes Cancer. 2010;1(7):700–7. 21103004 10.1177/1947601910382776 PMC2983490

[24] Zhou M, Cheng H, Fu Y, Zhang J. Long noncoding RNA DARS-AS1 regulates TP53 ubiquitination and affects ovarian cancer progression by modulation miR-194-5p/RBX1 axis. J Biochem Mol Toxicol. 2021;35(10): e22865. 34328246 10.1002/jbt.22865

[25] Bungsy M, Palmer MCL, Jeusset LM, Neudorf NM, Lichtensztejn Z, Nachtigal MW, et al. Reduced RBX1 expression induces chromosome instability and promotes cellular transformation in high-grade serous ovarian cancer precursor cells. Cancer Lett. 2021;500:194–207. 33290867 10.1016/j.canlet.2020.11.051

[26] Kunishige T, Migita K, Matsumoto S, Wakatsuki K, Nakade H, Miyao S, et al. Ring box protein-1 is associated with a poor prognosis and tumor progression in esophageal cancer. Oncol Lett. 2020;20(3):2919–27. 32782608 10.3892/ol.2020.11840 PMC7400995

[27] Zheng N, Schulman BA, Song L, Miller JJ, Jeffrey PD, Wang P, et al. Structure of the Cul1-Rbx1-Skp1-F boxSkp2 SCF ubiquitin ligase complex. Nature. 2002;416(6882):703–9. 11961546 10.1038/416703a

[28] Xie CM, Wei W, Sun Y. Role of SKP1-CUL1-F-box-protein (SCF) E3 ubiquitin ligases in skin cancer. J Genet Genomics. 2013;40(3):97–106. 23522382 10.1016/j.jgg.2013.02.001 PMC3861240

[29] Zhang W, Li L, Cai L, Liang Y, Xu J, Liu Y, et al. Tumor-associated antigen Prame targets tumor suppressor p14/ARF for degradation as the receptor protein of CRL2(Prame) complex. Cell Death Differ. 2021;28(6):1926–40. 33504946 10.1038/s41418-020-00724-5 PMC8184998

[30] Sarvari P, Rasouli SJ, Allanki S, Stone OA, Sokol AM, Graumann J, et al. The E3 ubiquitin-protein ligase Rbx1 regulates cardiac wall morphogenesis in zebrafish. Dev Biol. 2021;480:1–12. 34363825 10.1016/j.ydbio.2021.07.019

[31] Chen L, Yuan R, Wen C, Liu T, Feng Q, Deng X, et al. E3 ubiquitin ligase UBR5 promotes pancreatic cancer growth and aerobic glycolysis by downregulating FBP1 via destabilization of C/EBPalpha. Oncogene. 2021;40(2):262–76. 33122826 10.1038/s41388-020-01527-1

[32] Kannt A, Dikic I. Expanding the arsenal of E3 ubiquitin ligases for proximity-induced protein degradation. Cell Chem Biol. 2021;28(7):1014–31. 33945791 10.1016/j.chembiol.2021.04.007

[33] Sharma B, Saxena H, Negi H. Genome-wide analysis of HECT E3 ubiquitin ligase gene family in Solanum lycopersicum. Sci Rep. 2021;11(1):15891. 34354159 10.1038/s41598-021-95436-2 PMC8342558

[34] Liu M, Wang Y, Ruan Y, Bai C, Qiu L, Cui Y, et al. PKM2 promotes reductive glutamine metabolism. Cancer Biol Med. 2018;15(4):389–99. 30891326 10.20892/j.issn.2095-3941.2018.0122 PMC6420233

[35] Chiavarina B, Whitaker-Menezes D, Martinez-Outschoorn UE, Witkiewicz AK, Birbe R, Howell A, et al. Pyruvate kinase expression (PKM1 and PKM2) in cancer-associated fibroblasts drives stromal nutrient production and tumor growth. Cancer Biol Ther. 2011;12(12):1101–13. 22236875 10.4161/cbt.12.12.18703 PMC3335944

[36] Yoo SK, Song YS, Park YJ, Seo JS. Recent improvements in genomic and transcriptomic understanding of anaplastic and poorly differentiated thyroid cancers. Endocrinol Metab (Seoul). 2020;35(1):44–54. 32207263 10.3803/EnM.2020.35.1.44 PMC7090308

[37] Kuranaga Y, Sugito N, Shinohara H, Tsujino T, Taniguchi K, Komura K, et al. SRSF3, a splicer of the PKM gene, regulates cell growth and maintenance of cancer-specific energy metabolism in colon cancer cells. Int J Mol Sci. 2018;19(10):3012. 30279379 10.3390/ijms19103012 PMC6213643

[38] Toma-Fukai S, Shimizu T. Structural diversity of ubiquitin E3 ligase. Molecules. 2021;26(21):6682. 34771091 10.3390/molecules26216682 PMC8586995

[39] Xie Y, Liu YK, Guo ZP, Guan H, Liu XD, Xie DF, et al. RBX1 prompts degradation of EXO1 to limit the homologous recombination pathway of DNA double-strand break repair in G1 phase. Cell Death Differ. 2020;27(4):1383–97. 31562368 10.1038/s41418-019-0424-4 PMC7205894

