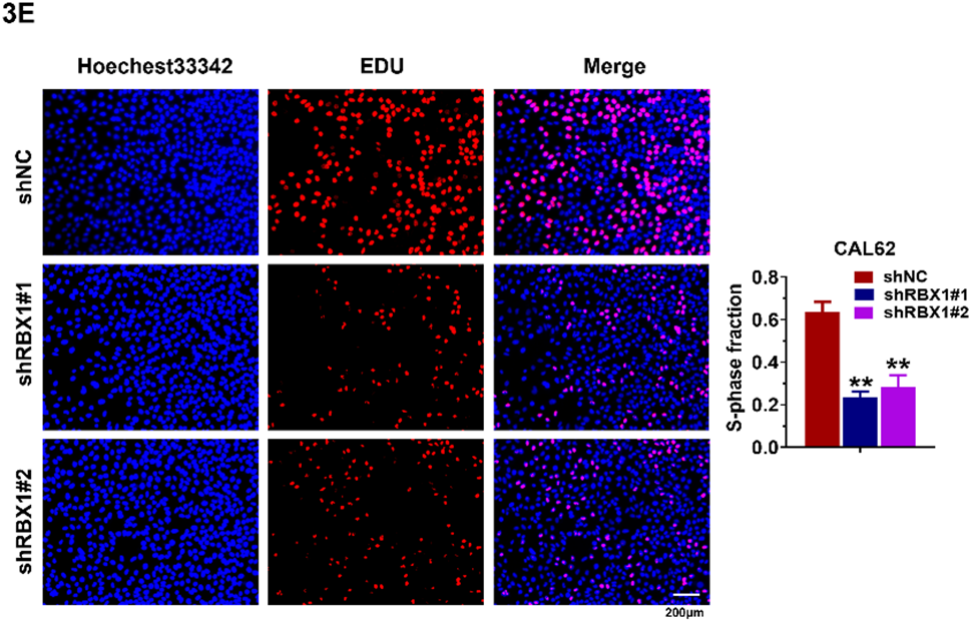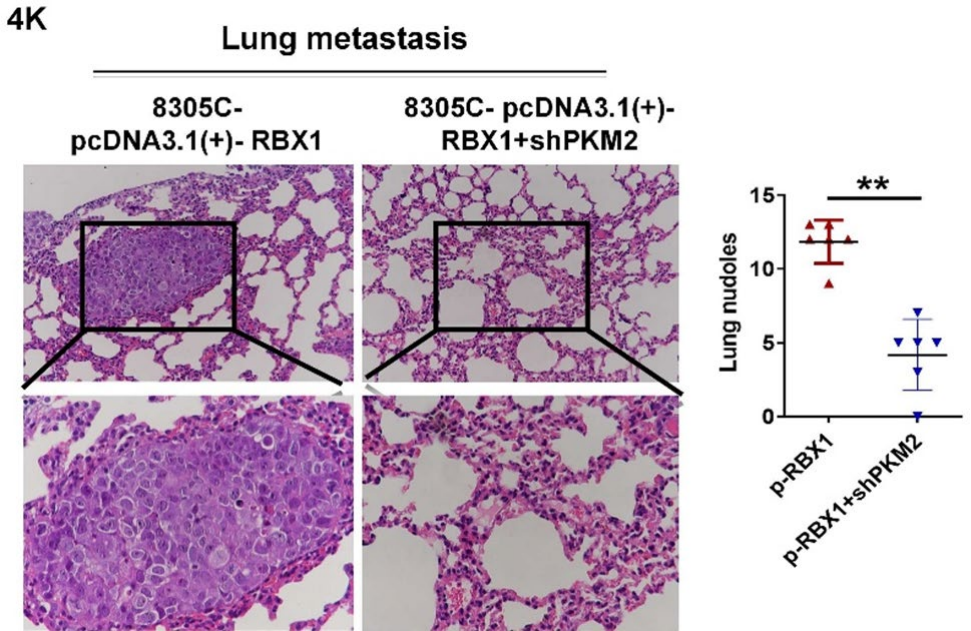# Correction: RBX1 regulates PKM alternative splicing to facilitate anaplastic thyroid carcinoma metastasis and aerobic glycolysis by destroying the SMAR1/HDAC6 complex

**DOI:** 10.1186/s13578-026-01560-9

**Published:** 2026-04-28

**Authors:** Debin Xu, Jichun Yu, Yuting Yang, Yunyan Du, Hongcheng Lu, Shouhua Zhang, Qian Feng, Yi Yu, Liang Hao, Jun Shao, Leifeng Chen

**Affiliations:** 1 Department of Thyroid Surgery, Second Affiliated Hospital of Nanchang University, No. 1 Minde Road, Nanchang, 330008 China; 2 Department of Intensive Care Unit, First Affiliated Hospital of Nanchang University, No. 17, Yongwai Main Street, Nanchang, 330006 China; 3 School of Pharmacy, Nanchang University, No. 471, Bayi Road, Nanchang, 330006 China; 4 Department of General Surgery, Second Affiliated Hospital of Nanchang University, No. 1 Minde Road, Nanchang, 330008 China; 5 Department of General Surgery, Affiliated Children’s Hospital of Nanchang University, No. 122, Yangming Road, Nanchang, 330006 China; 6 Department of Urology, Second Affiliated Hospital of Nanchang University, No. 1 Minde Road, Nanchang, 330008 China; 7 Department of Orthopaedics, Second Affiliated Hospital of Nanchang University, No. 1 Minde Road, Nanchang, 330008 China; 8 Department of Cardiovascular Surgery, Second Affiliated Hospital of Nanchang University, No. 1 Minde Road, Nanchang, 330008 China; 9 Cancer Center, Renmin Hospital of Wuhan University, No. 238 Jiefang Road, Wuhan, 430060 China

**Correction to**: Cell Biosci (2023) **13**:36. 10.1186/s13578-023-00987-8

In this article [1], the authors have noticed that two image file were inadvertently used or misplaced in Fig. 3E and Fig. 4K panels, likely due to processing a large number of images in the original data folder. The original article has been corrected.

The corrected figures (Fig. 3E and 4E) are given below.