# Supplementary figures and images for: RBX1 regulates PKM alternative splicing to facilitate anaplastic thyroid carcinoma metastasis and aerobic glycolysis by destroying the SMAR1/HDAC6 complex
Source: Cell Biosci. 2023 Feb 21;13:36. doi: 10.1186/s13578-023-00987-8 (PMC9945352; doi:10.1186/s13578-023-00987-8)

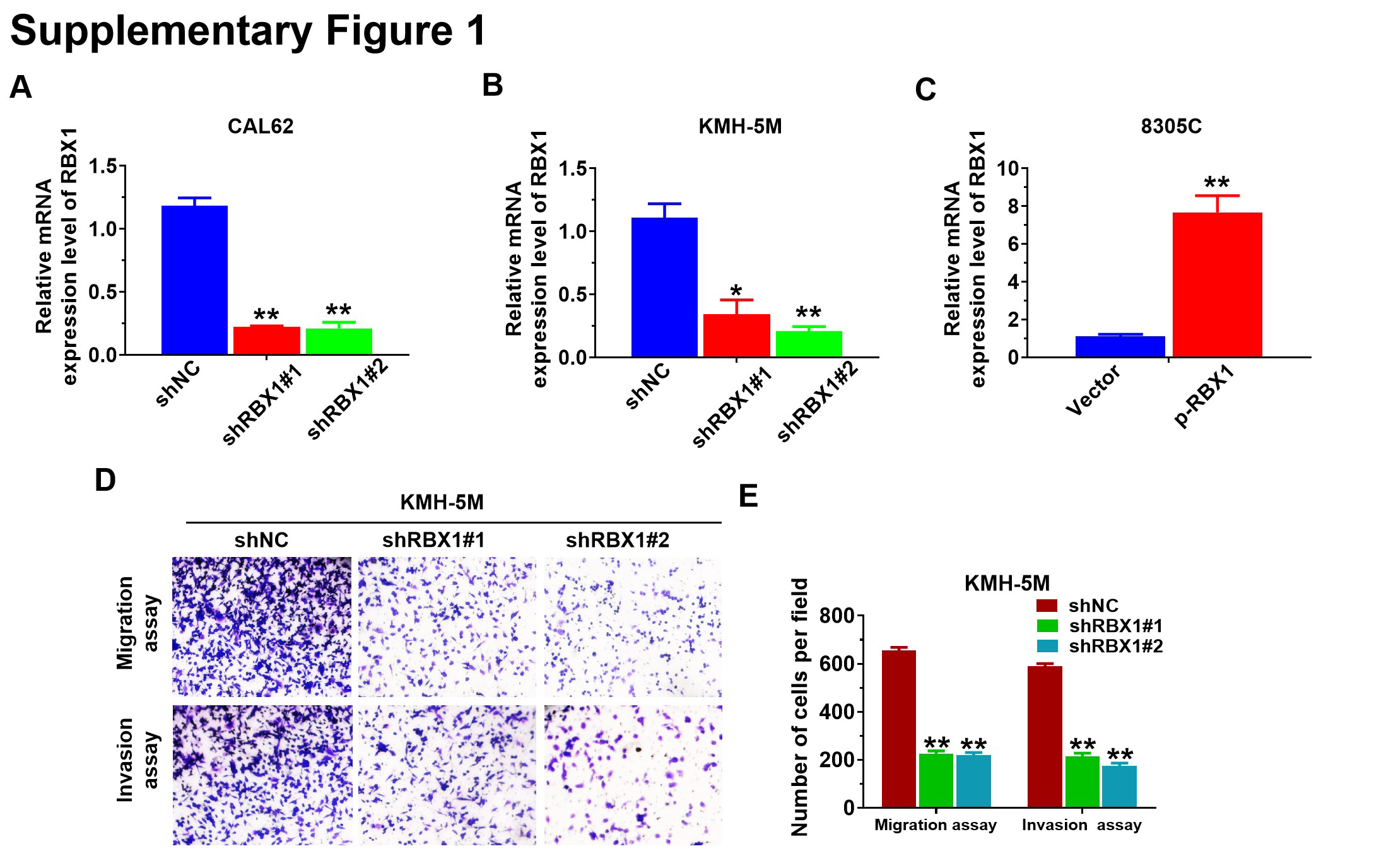

Supplement: Supplementary file 1 — Additional file 1: Figure S1. RBX1 promoted the migration of ATC cells. A-B. qRT-PCR was used to detect the expression levels of RBX1 in CAL62 and KMH-5M cells stably transfected with the shRBX1 plasmid. C. qRT-PCR was used to detect the expression levels of RBX1 in 8305C cells stably transfected with the HA-RBX1 plasmid. D-E. Transwell assays of KMH-5M cells transfected with shRBX1 plasmid. **P < 0.01. [file 13578_2023_987_MOESM1_ESM.tif]

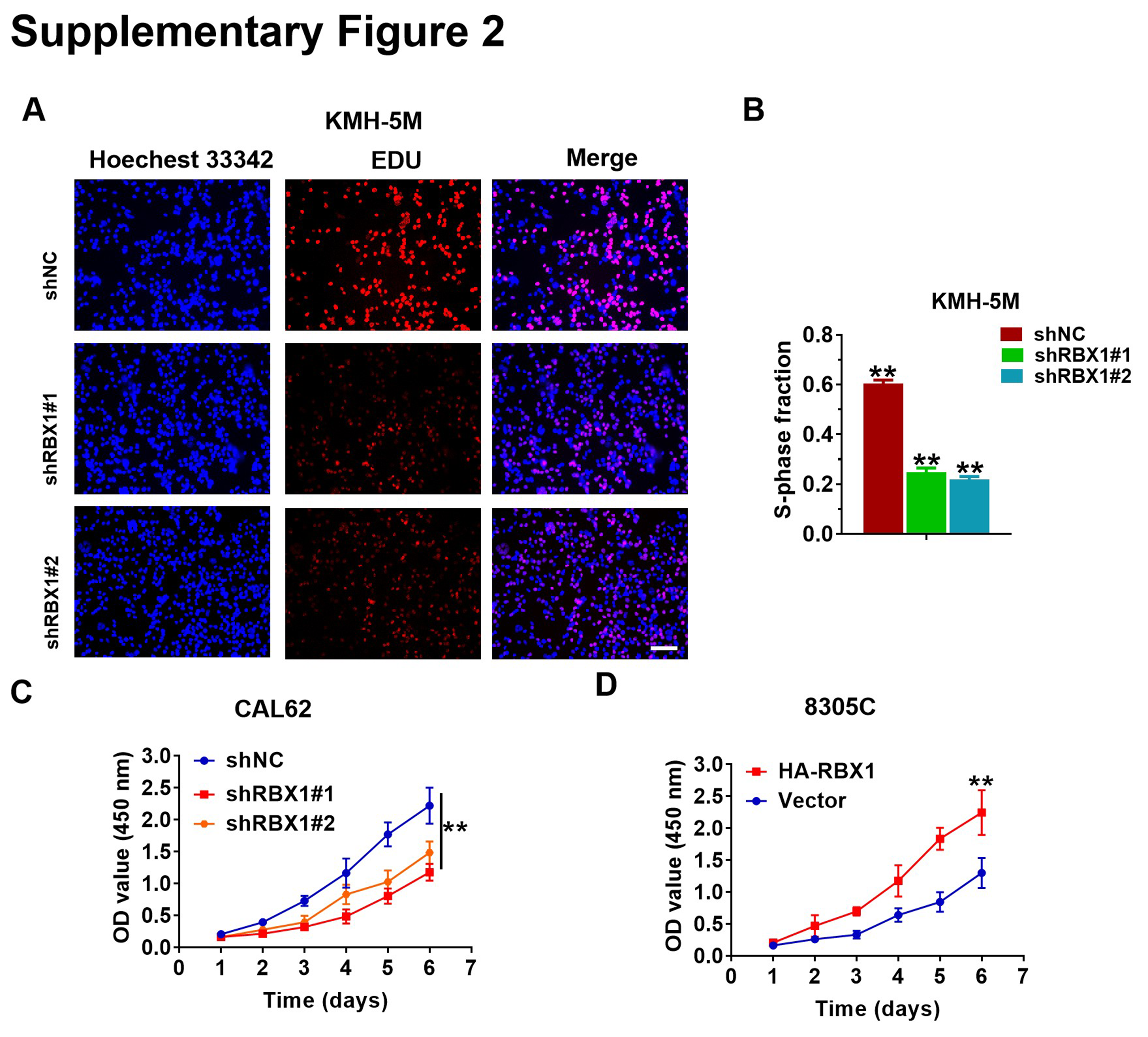

Supplement: Supplementary file 2 — Additional file 2: Figure S2. RBX1 promoted the proliferation of ATC cells. A, B. Edu assays of KMH-5M cells transfected with shRBX1 plasmid. **P < 0.01. C-D. CCK8 assays of CAL62 and 8305 cells transfected with shRBX1 plasmid or HA-RBX1 plasmid. [file 13578_2023_987_MOESM2_ESM.tif]

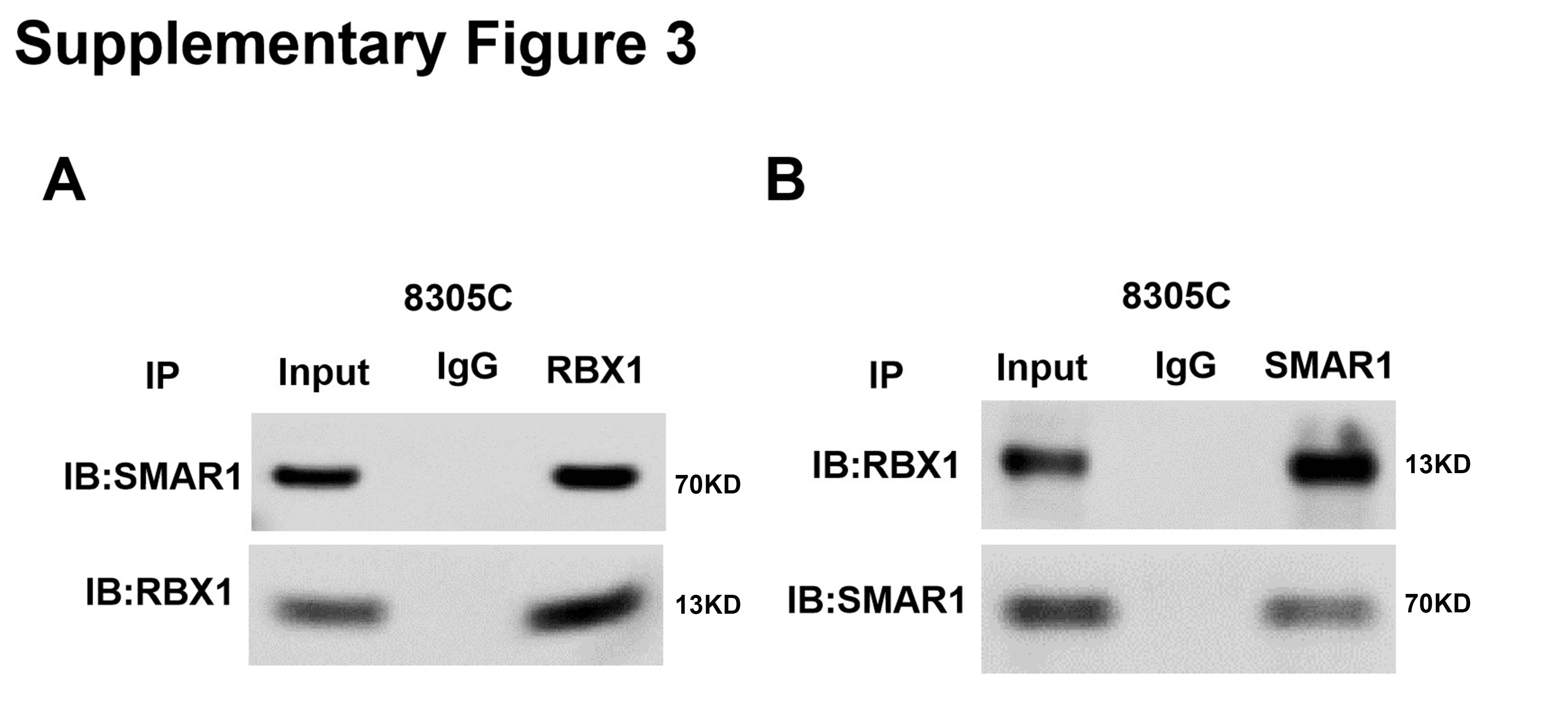

Supplement: Supplementary file 3 — Additional file 3: Figure S3. Co-IP showing direct binding of endogenous RBX1 and SMAR1. A-B.Co-IP showing direct binding of endogenous RBX1 and SMAR1 in 8305C cells. [file 13578_2023_987_MOESM3_ESM.tif]

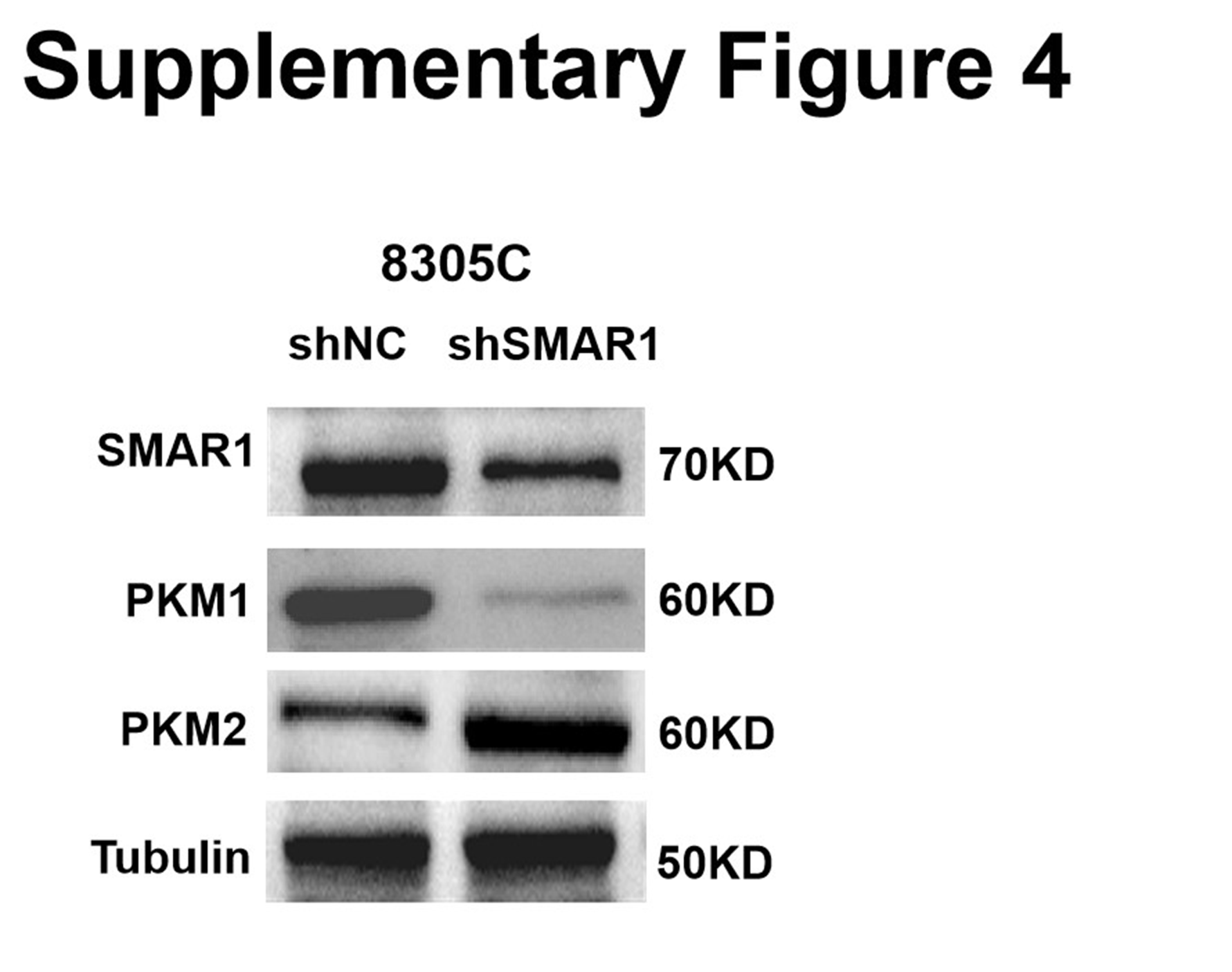

Supplement: Supplementary file 4 — Additional file 4: Figure S4. Expression of PKM isoforms upon shRNA-mediated knockdown of SMAR1 in 8305C by western blot. [file 13578_2023_987_MOESM4_ESM.tif]
