# Supplementary material for: RBX1 regulates PKM alternative splicing to facilitate anaplastic thyroid carcinoma metastasis and aerobic glycolysis by destroying the SMAR1/HDAC6 complex
Source: Cell Biosci. 2023 Feb 21;13:36. doi: 10.1186/s13578-023-00987-8 (PMC9945352; doi:10.1186/s13578-023-00987-8)
Supplement: Supplementary file 5 — Additional file 5: Table S1. RBX1-interacting proteins in ATC cells using mass spectrometry. [file 13578_2023_987_MOESM5_ESM.doc]

**Supplementary Table 1**

| **Gene names** | **MW [kDa]** | **Protein score** | **Sequence coverage (%)** | **# Unique Peptides** | **# Peptides** |
| --- | --- | --- | --- | --- | --- |
| MYH9 | 226.4 | 17214 | 62 | 121 | 139 |
| MYH10 | 228.9 | 10315 | 59 | 102 | 118 |
| ACTB | 41.7 | 6425 | 80 | 1 | 30 |
| ACTG1 | 41.8 | 6424 | 80 | 1 | 30 |
| MYH14 | 227.7 | 4622 | 45 | 70 | 79 |
| SMAR1 | 59 | 3601 | 43 | 73 | 73 |
| MYO6 | 149.6 | 2124 | 38 | 43 | 43 |
| ACTN4 | 104.8 | 2054 | 52 | 23 | 38 |
| GSN | 85.6 | 1803 | 37 | 24 | 24 |
| ACTA2 | 42 | 1634 | 44 | 5 | 19 |
| SPTAN1 | 284.4 | 1458 | 21 | 39 | 39 |
| HSPA9 | 73.6 | 1394 | 40 | 24 | 24 |
| PARP1 | 113 | 1333 | 39 | 31 | 31 |
| DBN1 | 71.4 | 1323 | 40 | 21 | 21 |
| CUL1 | 89.6 | 1272 | 38 | 25 | 25 |
| ACTBL2 | 42 | 1121 | 20 | 1 | 9 |
| CUL2 | 86.9 | 1066 | 34 | 23 | 23 |
| CUL3 | 88.9 | 1048 | 33 | 23 | 23 |
| CUL4B | 103.9 | 965 | 32 | 27 | 27 |
| TMOD3 | 39.6 | 923 | 54 | 15 | 17 |
| MYO1D | 116.1 | 897 | 32 | 26 | 26 |
| MYO5A | 215.3 | 855 | 17 | 22 | 26 |
| MYL6B | 22.8 | 835 | 58 | 13 | 15 |
| TPM4 | 28.5 | 825 | 56 | 14 | 19 |
| TPM3 | 32.9 | 812 | 45 | 7 | 15 |
